# From traditional remedy to modern therapy: a comprehensive review of palmatine’s multi-target mechanisms and ethnopharmacological potential

**DOI:** 10.3389/fphar.2025.1624353

**Published:** 2025-07-25

**Authors:** Zhoujing Shi, Lutuo Han, Huasen Yang, Geling Lu, Zhouying Shi, Boyan Ma

**Affiliations:** ^1^ College of Basic Medicine, Heilongjiang University of Chinese Medicine, Harbin, China; ^2^ Traditional Chinese Medicine Museum, Heilongjiang University of Chinese Medicine, Harbin, China; ^3^ Jiamusi Campus, Heilongjiang University of Chinese Medicine, Kiamusze, China; ^4^ College of Traditional Chinese Medicine, Changchun University of Chinese Medicine, Changchun, China

**Keywords:** palmatine, traditional botanical drug medicine, molecular mechanism, multiple target point, pharmacology, metabolism

## Abstract

**Background:**

As a core active metabolite of traditional Chinese medicines including *Coptis chinensis Franch.* and *Phellodendron amurense Rupr*., palmatine has been employed in Asian traditional medicine for centuries, primarily for treating jaundice, liver diseases, and inflammatory disorders. Modern research reveals that this isoquinoline alkaloid exhibits multi-target regulatory properties, demonstrating broad therapeutic potential across various diseases. This review systematically elucidates the molecular mechanisms of palmatine in treating digestive system disorders, neurological diseases, metabolic diseases, cancer and so on and cancers, with particular emphasis on analyzing its “disease-target-pathway” relationships.

**Methods:**

In accordance with the PRISMA guidelines, a comprehensive literature search was conducted using PubMed, Web of Science, and Embase databases. The search terms included “Palmatine,” “Disease,” “*in vitro*/*in vivo* experiment,” “Inflammation,” “Anti-inflammatory,” and “Antioxidation.” among others. The search covered all English-language articles related to palmatine published between 1 January 2014, and 31 May 2025. All included studies underwent a quality assessment.

**Results:**

Studies demonstrate palmatine’s multi-target mechanisms through regulation of NF-κB/NLRP3, Nrf2/HO-1, and AMPK/mTOR signaling pathways, mediating anti-inflammatory, antioxidant, and metabolic-modulating effects. Its exceptional blood-brain barrier permeability confers distinct advantages for central nervous system disorders, while its metabolites such as 8-oxypalmatine display superior bioactivity. In anticancer applications, palmatine functions through multiple mechanisms encompassing direct tumor cell cytotoxicity, metastasis suppression, and angiogenesis inhibition, while maintaining dose-dependent safety characteristics.

**Conclusion:**

Palmatine serves as a bridge connecting traditional medicine and modern therapy, offering novel strategies for complex diseases through its polypharmacological actions. Although limited by low bioavailability, clinical potential can be enhanced via combination therapies, structural modifications such as C13 alkylation, and nano-delivery systems. Future research should prioritize exploration of synergistic effects, targeted delivery technologies, and large-scale clinical validation.

## Graphical Abstract

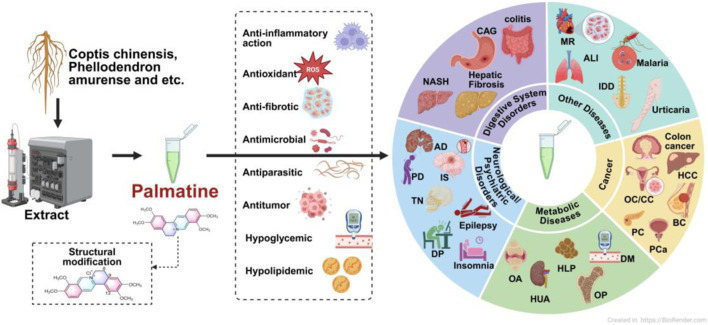

## 1 Introduction

Palmatine, a naturally occurring isoquinoline alkaloid, represents a pharmacologically significant metabolite of traditional medicinal plants such as *Coptis chinensis Franch*. and *Phellodendron amurense Rupr*. As early as recorded in the medicinal plants were documented as early as in *Shennong Bencao Jing*, the earliest extant Chinese pharmacopoeia, “*Coptis chinensis Franch* governs heat syndromes, eye pain. intestinal inflammation, abdominal pain, and diarrhea”, being used to treat inflammatory diseases (“heat syndrome”, diarrhea). The *Compendium of Materia Medica* also elaborately describes its therapeutic effects on diseases such as jaundice and dysentery. Modern research has confirmed its anti-inflammatory and antioxidant properties.

Modern research has further confirmed the anti-inflammatory and antioxidant properties of palmatine. Recent studies demonstrate that palmatine’s therapeutic effects primarily arise from its regulation of two classical pathways: anti-inflammatory and antioxidant activities. Specifically, palmatine exerts its anti-inflammatory effects by inhibiting NLRP3 inflammasome activation and NF-κB signaling (Mai et al., 2019; Pereira et al., 2023), while combating oxidative stress through activation of the nuclear factor erythroid 2-related factor 2 (Nrf2)/heme oxygenase-1 (HO-1) pathway (Cheng et al., 2022a; Pei et al., 2023; Wang et al., 2023; Zuo et al., 2024). In metabolic regulation, palmatine restores glucose and lipid homeostasis via the AMP-activated protein kinase (AMPK)/mTOR and insulin receptor substrate 1 (IRS1)/RAC-β serine/threonine-protein kinase (AKT2)/forkhead box protein O1 (FOXO1)/glucose transporter type 2 (GLUT2) pathways (Chen et al., 2021; Lin et al., 2022; Nwabueze et al., 2022; Xia et al., 2022), providing a mechanistic explanation for its traditional use in metabolic disorders such as diabetes. Notably, palmatine’s ability to cross the blood-brain barrier and modulate the gut-liver axis further distinguishes it as a multi-target therapeutic agent for neurological and digestive system disorders (Kiris et al., 2023; Qin et al., 2023).

Despite these advances, there remains a lack of systematic analysis regarding its primary mechanisms in disease treatment. This review synthesizes evidence from the past decade to elucidate palmatine’s core pharmacological network, systematically summarizing its molecular mechanisms in digestive, neurological, and metabolic diseases. By incorporating recent perspectives on palmatine’s pharmacokinetics, pharmacological effects, safety, and toxicity, we aim to better reveal its therapeutic potential, fill gaps in existing reviews, and bridge traditional wisdom with modern therapeutic strategies, thereby providing references for future research and clinical applications.

## 2 Search strategy and selection criteria

### 2.1 Search strategy

A comprehensive literature search was conducted in accordance with PRISMA guidelines using PubMed, Web of Science, and Embase databases. The search encompassed all English-language articles related to “palmatine” published between 1 January 2014, and 31 May 2025. The search terms included: “Palmatine,” “Disease,” “*in vitro*/*in vivo* experiment,” “Inflammation,” “Anti-inflammatory,” “Antioxidation,” “Anti-fibrosis,” “Tumor,” “Metabolism,” “Neurological diseases,” “Digestion,” “Pharmacokinetics,” “Pharmacology,” “Colitis,” “Chronic Atrophic Gastritis,” “Liver Diseases,” “Hepatic Fibrosis,” “Alzheimer’s Disease,” “Parkinson’s Disease,” “Ischemic Stroke,” “Trigeminal Neuralgia,” “Epilepsy,” “Depression,” “Insomnia,” “Diabetes,” “Hyperlipidemia,” “Hyperuricemia and Osteoarthritis,” “Osteoporosis,” “Cancer,” “Tumor,” “Myocardiopathy,” “Lung Injury,” “Malaria,” “Intervertebral Disc Degeneration,” and “Urticaria.”

### 2.2 Literature inclusion and exclusion criteria

The inclusion criteria were as follows: (1) studies that explicitly identified palmatine as the primary active metabolite and main research subject; (2) experimental studies focusing on palmatine’s therapeutic effects in disease treatment, limited to original research articles; (3) studies exploring the molecular mechanisms or signaling pathways of palmatine’s pharmacological actions; (4) research reporting specific mechanisms or potential therapeutic targets of palmatine in disease treatment; and (5) studies with well-defined experimental designs, appropriate control groups, complete datasets, and rigorous statistical analyses.

Exclusion criteria comprised: (1) studies lacking clear descriptions of palmatine’s therapeutic mechanisms or disease targets; (2) literature with insufficient methodological details regarding palmatine’s study design, methodology, or mechanistic investigations; (3) studies with low methodological quality or questionable reliability of results; (4) duplicate publications or studies with substantial content overlap; and (5) articles with unavailable full texts or incomplete data.

Literature Screening Process: Two independent researchers (ZS and LH) performed the literature screening. Initially, titles and abstracts were reviewed for preliminary selection, followed by full-text assessment to determine final inclusion. Any discrepancies were resolved through discussion or adjudication by a third researcher. The screening process involved cataloging publication years, titles, authors, botanical sources of palmatine, purity or extraction methods, experimental models, drug concentrations and dosages, and mechanistic data. All included studies underwent independent evaluation and analysis to ensure the reliability of the findings.

## 3 Physicochemical properties and In vivo fate of palmatine

### 3.1 Structural characteristics and natural sources

**FIGURE 1:**
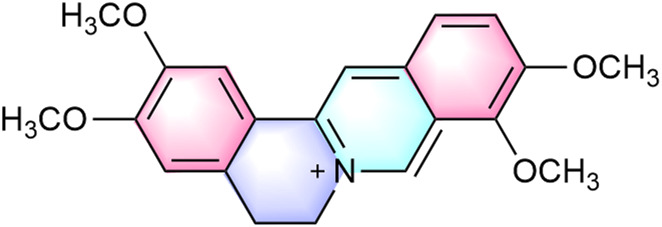
Chemical structure of palmatine.

Palmatine is a natural isoquinoline alkaloid belonging to the quaternary protoberberine class, commonly found in various medicinal plants (Ekeuku et al., 2020). It primarily occurs in the rhizomes of: *Berberidaceae* (e.g., *Phellodendron amurense Rupr*., *Berberis sibirica Pall.), Papaveraceae* (e.g., *Corydalis yanhusuo* [Y. H. Chou and Chun C. Hsu] W. T. Wang ex Z. Y. Su and C. Y. Wu), *Ranunculaceae* (e.g., *Coptis chinensis Franch.*, *Thalictrum glandulosissimum* [Finet and Gagnep.] W. T. Wang and S. H. Wang), and *Menispermaceae* (e.g., *Fibraurea recisa Pierre*, *Stephania japonica* var. *hispidula Yamam*.) (Kiris et al., 2023; Xu et al., 2024; Zeng J. et al., 2024). Notably, the highest concentrations occur in plant roots (Chacón-Fuentes et al., 2025). These plants also contain structurally related metabolites, including berberine, tetrahydroberberine, dehydrocorydaline, magnoflorine, worenine, and coptisine (Gao et al., 2022; Liu et al., 2016). As a quaternary ammonium salt, palmatine (molecular formula: C_21_H_22_NO_4_^+^) exhibits distinct structural features conferring diverse bioactivities. Its molecular structure contains two methoxy groups at the C2 and C3 positions of the aromatic ring (Ekeuku et al., 2020) (Figure 1). The donor-acceptor system comprises a conjugated aromatic ring and an isoquinoline core, exhibiting π-π interactions that facilitate weak charge-transfer emission states, explaining its unique photophysical properties and solid-state luminescence (Xu et al., 2021). Recent studies indicate palmatine may disrupt double-stranded TRF2 sequences, promoting G-quadruplex formation in G-rich regions (Fazelifar et al., 2025). This mechanism is of particular interest in oncology, as G-quadruplex stabilization in proto-oncogene regulatory regions represents a potential anticancer strategy.

In traditional medical systems, palmatine, the predominant bioactive metabolite of medicinal plants, has been extensively employed across Asian therapeutic practices for treating jaundice, hepatic disorders, hypertension, and diverse inflammatory conditions including tonsillitis, enteritis, urinary tract infections, and gynecological inflammations. Modern studies have confirmed that these traditional applications are closely associated with palmatine’s diverse pharmacological effects, including anti-inflammatory, antibacterial, sedative, analgesic, and hypnotic activities (Li et al., 2015; Ma et al., 2021; Ma W. K. et al., 2016; Tarabasz and Kukula-Koch, 2020). Although palmatine demonstrates significant therapeutic potential for various modern diseases, its chemical structure may partially limit its transport capacity via human organic cation transporter 1 (hOCT1), resulting in relatively low bioavailability that restricts clinical application to some extent. This also partly explains why traditional preparations often require high doses. However, contemporary research suggests that palmatine may indirectly enhance the cellular uptake and distribution of other alkaloids through interactions with human organic cation transporter 2 (hOCT2), thereby improving overall bioavailability (Zhang et al., 2022).

### 3.2 Absorption and distribution characteristics

Isoquinoline alkaloids generally exhibit low bioavailability. Animal studies demonstrate that the absolute bioavailability of orally administered palmatine in rats, as calculated, does not exceed 10% (Song et al., 2021). In pharmacokinetic experiments, Sprague-Dawley rats received palmatine at oral doses of 10, 30, and 60 mg/kg or intravenous administration of 2.5 mg/kg. Comparative analysis revealed maximum plasma concentrations (C_max_) of 86 ± 10 ng/mL (10 mg/kg), 81 ± 39 ng/mL (30 mg/kg), and 273 ± 168 ng/mL (60 mg/kg) for oral administration, significantly lower than the intravenous C_max_ of 397 ± 140 ng/mL (Song et al., 2021).

Pharmacokinetic parameters further indicated that intravenous palmatine administration resulted in faster absorption and slower elimination compared to oral dosing. The time to reach maximum concentration (T_max_) was 0.1 ± 0.0 h for intravenous injection versus 0.9 ± 0.9 h (10 mg/kg), 1.3 ± 0.5 h (30 mg/kg), and 0.6 ± 0.4 h (60 mg/kg) for oral administration. Corresponding elimination half-lives (t_1/2_) were 23.3 ± 14.0 h (intravenous) compared to 5.7 ± 2.1 h (10 mg/kg), 5.6 ± 0.82 h (30 mg/kg), and 3.8 ± 0.7 h (60 mg/kg) for oral dosing. Notably, the apparent volume of distribution was substantially higher following intravenous administration (95.5 ± 47.1 L/kg) than oral dosing (28.4 ± 18.7, 24.8 ± 8.9, and 17.1 ± 8.2 L/kg) (Song et al., 2021), suggesting more extensive tissue distribution. This pharmacokinetic profile may be attributed to poor intestinal absorption, significant hepatic and intestinal first-pass effects, and metabolite interference (Zhang et al., 2022).

Studies indicate that palmatine clearance remains independent of administration route and shows no dose-dependent variation. No significant differences were observed between oral and intravenous clearance (CL) values: 3.2 ± 1.2 L/h/kg (oral, 10 mg/kg), 3.0 ± 1.1 L/h/kg (oral, 30 mg/kg), 3.1 ± 1.3 L/h/kg (oral, 60 mg/kg), and 3.1 ± 1.2 L/h/kg (IV, 2.5 mg/kg). Plasma concentrations decreased markedly by 8 h post-administration, demonstrating no long-term accumulation (Song et al., 2021). However, palmatine plasma protein binding exhibits concentration-dependent decreases. In rat plasma, binding rates were 71.13% ± 0.49% (1.0 μg/mL), 51.17% ± 0.39% (4.0 μg/mL), and 41.81% ± 0.74% (10.0 μg/mL). Human plasma demonstrated higher binding: 74.43% ± 0.09% (1.0 μg/mL), 74.34% ± 0.09% (4.0 μg/mL), and 55.50% ± 0.54% (10.0 μg/mL) (Song et al., 2021). This likely reflects limited plasma protein binding sites, where saturation leads to reduced binding at higher concentrations. The observed species difference in plasma protein binding (human > rat) warrants particular attention during drug interaction studies and clinical applications to ensure therapeutic safety and efficacy.

Animal studies demonstrate that after oral administration of 30 mg/kg palmatine to rats, the metabolite becomes detectable in multiple tissues including heart, liver, spleen, lungs, kidneys, brain, stomach, duodenum, jejunum, and ileum. The highest concentrations occur in gastrointestinal tissues, particularly the ileum. Significant renal accumulation is also observed, likely related to renal excretion pathways (Song et al., 2021). Additional research confirms palmatine’s notable hepatoprotective and nephroprotective effects through inhibition of oxidative stress and apoptosis (Khaksari et al., 2021). Quantitative structure-activity relationship (QSAR) studies and multiple reaction monitoring (MRM) analyses verify palmatine’s ability to effectively cross the blood-brain barrier and achieve therapeutic concentrations in brain tissue (Gawel et al., 2020; Kiris et al., 2023). This property provides a crucial pharmacological basis for its application in central nervous system disorders. Compared to most drugs with limited blood-brain barrier permeability, palmatine demonstrates unique advantages for neurological disease prevention and treatment.

Recent methodological advances employing near-infrared spectroscopy (NIR) combined with partial least squares regression (PLSR), particularly through competitive adaptive reweighted sampling (CARS) algorithms for characteristic wavelength extraction, have successfully established quantitative analytical models for palmatine content determination (Li et al., 2025). This approach overcomes the operational complexity and prolonged detection times associated with traditional high-performance liquid chromatography (HPLC) methods, enabling rapid and accurate quantification of palmatine concentrations. The technique facilitates precise assessment of absorption and distribution characteristics, supports content control for optimized drug formulation development, and provides both technical support for novel palmatine-based drug development and scientific basis for quality control and clinical application of traditional Chinese medicines.

### 3.3 Metabolic transformation and elimination pathways

**FIGURE 2:**
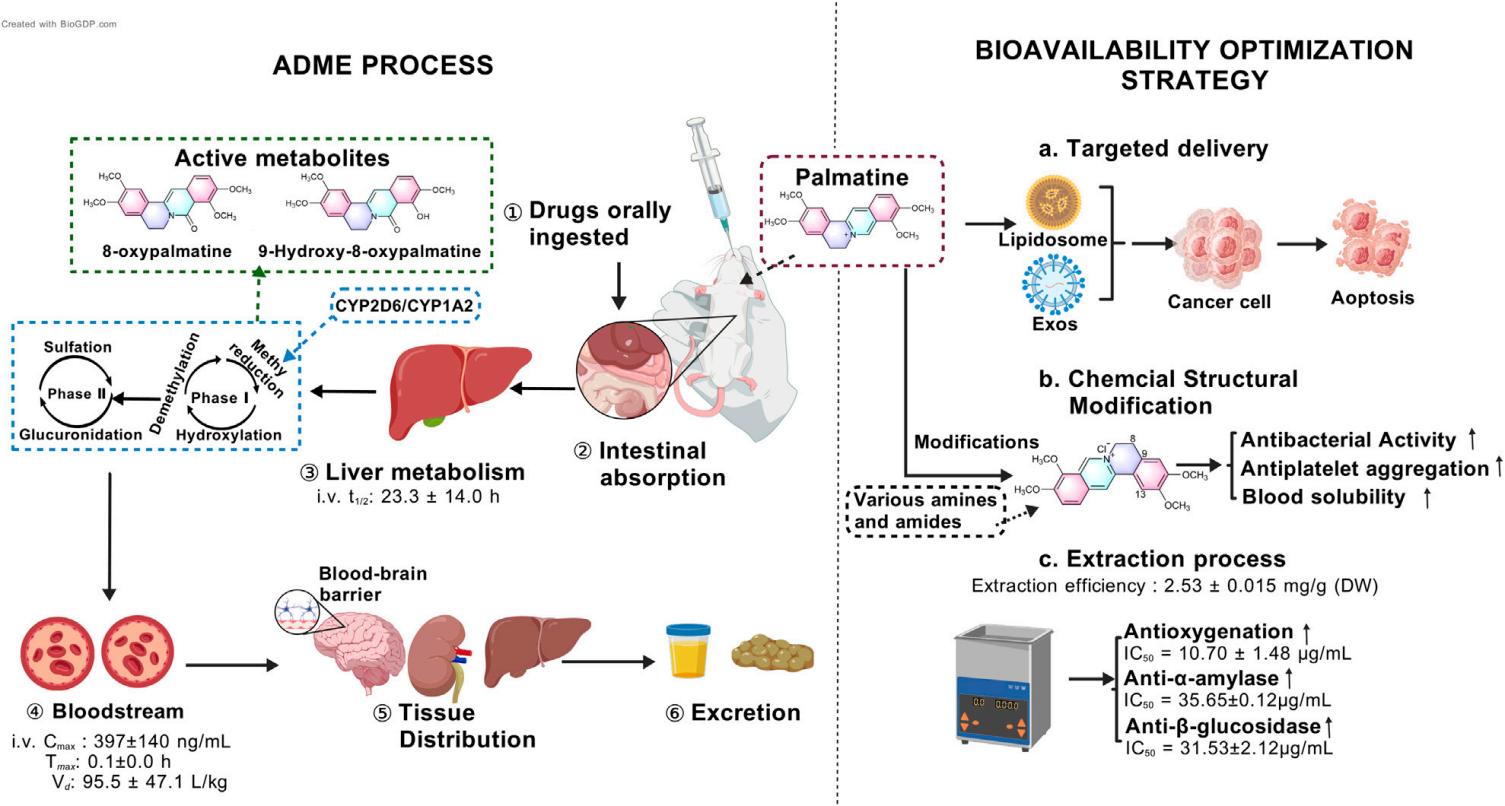
Multi-phase pharmacokinetic journey of palmatine and strategies to overcome its low bioavailability.

The metabolic pathways of palmatine primarily involve phase I reactions, including demethylation, hydroxylation, and methyl reduction (Li et al., 2017), as well as phase II reactions such as glucuronidation and sulfation. Its metabolites are widely distributed in urine, plasma, bile, liver, and feces (Wang et al., 2017). Recent studies further reveal that palmatine undergoes bioactivation in human hepatocytes via O-demethylation or hydroxylation, while exhibiting relative resistance to metabolic activity in both primary human hepatocytes and recombinant Cytochrome P450 (CYP) enzymes. Notably, O-demethylation of palmatine is mediated to a minor extent by human recombinant CYP2D6 and CYP1A2 (Vrba et al., 2015) (Figure 2).

### 3.4 Strategies for bioavailability enhancement

Recent research has focused on optimizing extraction techniques, developing novel biopharmaceutical formulations, and designing advanced delivery systems to improve palmatine’s solubility, stability, and targeting efficacy, thereby enhancing its bioavailability (Qi et al., 2025; Singh et al., 2025; Zeng et al., 2024a) (Figure 2). Studies demonstrate that hydrochloric acid/methanol-ultrasonic extraction of fresh *Phellodendron amurense Rupr.* bark yields palmatine at 1.25 mg/g (Xu et al., 2018), with favorable aqueous solubility (Xu et al., 2021). Singh et al. employed natural deep eutectic solvents (NADES) coupled with ultrasound-assisted extraction, achieving a palmatine yield of 2.53 ± 0.015 mg/g dry weight (DW). Subsequent purification via AB-8 macroporous resin yielded a recovery rate of 66.34% ± 0.77%. This optimized protocol not only improved extraction efficiency but also enhanced bioactivity: the NO• radical scavenging capacity (IC_50_ = 10.70 ± 1.48 μg/mL) surpassed that of ascorbic acid (IC_50_ = 24.67 ± 1.24 μg/mL) by 2.3-fold, while α-amylase and β-glucosidase inhibition reached IC_50_ values of 35.65 ± 0.12 μg/mL and 31.53 ± 2.12 μg/mL, respectively (Figures 2c). Notably, NADES, is composed of glycerol, glucose, citric acid, malic acid, or tartaric acid, reduced environmental toxicity compared to conventional organic solvents, aligning with sustainable development goals (Singh et al., 2025).

Capitalizing on palmatine’s aggregation-induced emission (AIE) properties, researchers found that concentrations as low as 80 μM effectively disrupt *Listeria* monocytogenes biofilms during photodynamic therapy, suggesting potential applications in food preservation (Peng et al., 2024). Targeted delivery strategies have emerged as pivotal for bioavailability improvement. Nanocarrier systems, including liposomes and polymeric carriers, facilitate the conjugation of peptide drugs. For example, modifications at the O-9 position can be carried out using lipophilic or targeting moieties. For instance, palmatine-antimicrobial peptide conjugates or exosome-based livery systems enhance tumor accumulation, prolong half-life, mitigate systemic distribution, and improve immune cell activation (Zeng et al., 2024b; Zhao et al., 2023) (Figures 2a). These techniques can not only increase the bioavailability of palmatine, but also enhance its tissue-specific distribution and reduce side effects.

In recent years, chemical structure modification and derivative development based on palmatine have provided novel strategies for improving its pharmacokinetic properties (Zeng Z. et al., 2024) (Figures 2b). Beyond the aforementioned physical methods to enhance palmatine’s antibacterial efficacy, researchers have also modified its chemical structure by introducing diverse functional groups at specific positions such as C-13 and C-8 to augment its interaction capacity with biological targets. For instance, quaternary 13-hexanoylpalmatine chloride, a derivative structurally modified by elongating the alkyl chain, exhibited a minimum inhibitory concentration (MIC) of 62.5 μg/mL against *Staphylococcus aureus*, significantly lower than that of unmodified palmatine (Song et al., 2018). Other studies introduced 2,4-dimethoxybenzylamine at C-13 or benzylamine groups including variants with diverse substituents at C-9 to enhance antibacterial activity (Fan et al., 2020). Regarding the improvement of other pharmacological activities, the introduction of a n-propyl ether moiety at C-9 of palmatine’s scaffold conferred potent antiplatelet aggregation properties (Fan J. H. et al., 2022). Furthermore, in structural optimization efforts, a carbodiimide-mediated condensation reaction with salicylic acid derivatives facilitated the design and synthesis of a novel palmatine derivative, 2q. This compound’s elevated topological molecular polar surface area (TPSA, 80.34 Å^2^) likely improves solubility, promoting blood diffusion prior to blood-brain barrier penetration (Pang et al., 2025). These advancements in modern biopharmaceutical development not only broaden the clinical applicability of palmatine but also establish a foundation for its utilization in precision medicine. Future research should focus on improving palmatine’s bioavailability through exploration of alternative protein molecular mechanisms, chemical structure optimization, and selection of appropriate dosage forms.

## 4 Molecular mechanisms of palmatine in disease pathogenesis

**FIGURE 3:**
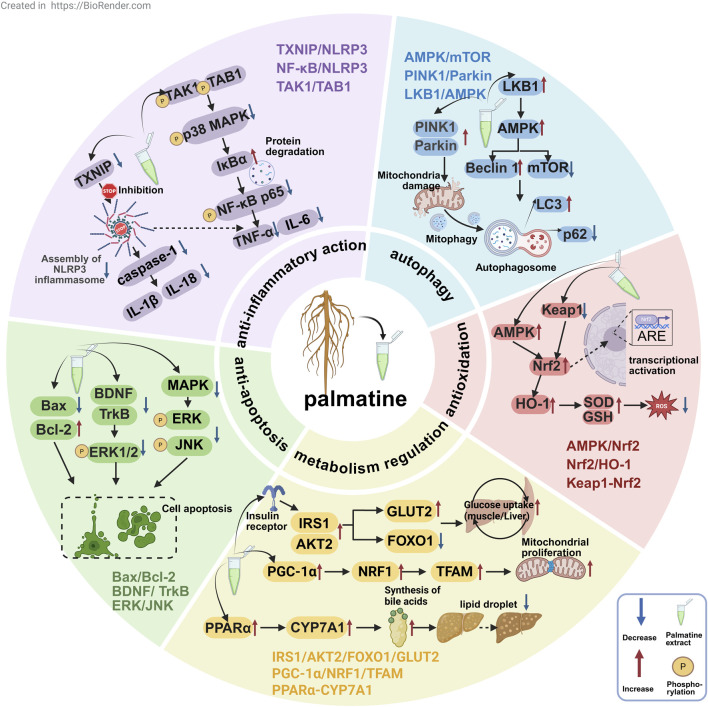
The mechanisms of palmatine in anti-inflammatory, antioxidant, autophagic, metabolic-regulatory, and anti-apoptotic pathways for multi-target disease therapy.

As a multi-target isoquinoline alkaloid, palmatine demonstrates unique therapeutic advantages through its modulation of key pathways including anti-inflammatory, antioxidant, autophagy, metabolic regulation, and anti-apoptotic mechanisms (Figure 3). This section systematically elaborates on palmatine’s mechanisms of action in digestive system disorders, neurological diseases, metabolic diseases, and cancer treatment. These findings not only validate the scientific basis of traditional medical applications but also provide novel insights for developing natural product-based, multi-target therapeutic strategies.

### 4.1 Digestive system disorders

#### 4.1.1 Intestinal inflammation and barrier repair

##### 4.1.1.1 Colitis

Colitis is a chronic inflammatory disease, and emerging evidence suggests that palmatine is a promising therapeutic agent for colitis through multifaceted mechanisms. As a bioactive alkaloid, palmatine serves as a potential immunomodulator for ulcerative colitis, exhibiting immunomodulatory, anti-inflammatory, and gastroprotective effects. It interacts with janus kinase 3 (JAK3), programmed cell death protein 1(PD-1), and PD-L1 to suppress the production of inflammatory cytokines in RAW 264.7 cells and inhibit signal transducer and activator of transcription 3 (STAT3) activation (Deng et al., 2022). Crucially, palmatine directly suppresses indoleamine 2,3-dioxygenase-1 (IDO-1) protein expression, with molecular docking studies confirming its high affinity for the IDO-1 catalytic pocket. By attenuating tryptophan catabolism, it reduces the generation of kynurenine and 5-hydroxytryptophan (5-HTP), pro-inflammatory kynurenine pathway metabolites, thereby promoting the restoration of mucosal immune homeostasis (Zhang et al., 2018a). In colitis models, palmatine exerts anti-inflammatory effects by facilitating the accumulation of tolerogenic dendritic cells (DCs) and enhancing regulatory T cell (Treg) differentiation (Huang et al., 2024). Additionally, it is associated with PTEN-induced Putative Kinase 1 (PINK1)/Parkin-mediated autophagy activation, protecting mice from dextran sulfate sodium (DSS)-induced damage via suppression of the nod-like receptor protein 3 (NLRP3) inflammasome (Mai et al., 2019). This process also involves inhibition of fat mass and obesity-associated protein (FTO) expression and modulation of m6A methylation (Ji et al., 2024).

Contemporary studies have demonstrated that palmatine ameliorates colitis-associated symptoms through multi-target mechanisms. It significantly upregulates methyltransferase-like 3 (METTL3) and METTL14 expression while downregulating AlkB homolog 5 (ALKBH5) and FTO expression, thereby modulating m6A methylation and suppressing the release of inflammatory cytokines, including tumor necrosis factor-α (TNF-α), IL-6, IL-8, and IL-1β. Furthermore, palmatine enhances the expression of tight junction protein zonulin-1 (ZO-1) in intestinal epithelial cells and increases transepithelial electrical resistance, consequently controlling inflammatory responses and restoring intestinal barrier function (Ji et al., 2024). These effects protect against dextran sulfate sodium (DSS)-induced intestinal damage by maintaining barrier integrity (Li et al., 2024). This mechanism not only blocks pathogens and toxins but, more importantly, reshapes the intestinal microenvironment and alleviates gut dysbiosis (Zhang X. J. et al., 2018). The restoration of gut microbiota homeostasis directly influences ion transport function in intestinal epithelial cells. Through this gut barrier-microbiota interaction mechanism, palmatine also exerts antidiarrheal effects via precise regulation of Ca^2+^ and K^+^ channels (Wu et al., 2008).

##### 4.1.1.2 Chronic Atrophic Gastritis

As a traditional antimicrobial agent, modern research on palmatine has focused on its inhibitory effects against *H. pylori* (*Helicobacter pylori*), which is closely associated with the prevention and treatment of chronic atrophic gastritis (CAG). Rong Q et al. demonstrated through *in vivo* and *in vitro* studies that palmatine exhibits dose-dependent antibacterial activity against *H. pylori* (Rong et al., 2016). Its unique mechanism involves specific inhibition of *H. pylori* urease (HPU, IC_50_ = 0.53 ± 0.01 mM) and jack bean urease (JBU, IC_50_ = 0.03 ± 0.00 mM), with potency comparable to the classical urease inhibitor acetohydroxamic acid. Molecular docking studies reveal that this inhibition targets sulfhydryl groups in the enzyme’s active center via N-H∙π interactions rather than direct binding to the active-site Ni^2+^, thereby stabilizing the inactive conformation of the enzyme (Zhou et al., 2017).

Beyond *H. pylori* eradication, palmatine protects gastric mucosa through multiple pathways. It attenuates matrix metalloproteinase 10 (MMP-10) -dependent inflammatory responses by suppressing the a disintegrin and metalloprotease 17 (ADAM17)/epidermal growth factor receptor (EGFR) signaling pathway, reducing H. pylori-induced histological damage to gastric mucosa and morphological alterations in gastric epithelial cells (GES-1) (Chen et al., 2020a). Additionally, it inhibits oxidative stress and inflammation via the STAT1/CXCL10 axis, modulating IL-17, TNF-α, and p-p65 expression to alleviate N-methyl-N′-nitro-N-nitrosoguanidine (MNNG)-induced CAG (Zhou et al., 2024). Metabolomic studies further indicate that palmatine systematically improves the gastric mucosal metabolic microenvironment by remodeling the tricarboxylic acid (TCA) cycle and interconnecting multiple metabolic pathways, including taurine and hypotaurine metabolism, glycerophospholipid metabolism, and pentose and glucuronate interconversions (Chen et al., 2020b). This multi-level mechanism—spanning pathogen clearance to mucosal protection—positions palmatine as a promising multi-target therapeutic agent for CAG.

#### 4.1.2 Hepatocyte injury and fibrosis

##### 4.1.2.1 Liver diseases

Palmatine exerts significant hepatoprotective effects by modulating the balance between autophagy and apoptosis. In an alcohol-induced liver injury model, palmatine (150 μg/mL) transiently induced Light Chain 3II (LC3-II) conversion and p62 degradation while upregulating the expression of autophagy-related genes ATG5 and ATG7 in ethanol-treated mouse hepatocytes, compared to both model and control groups. Concurrently, it inhibits the apoptotic pathway, as manifested by significantly increased expression of the anti-apoptotic protein B-cell lymphoma-2 (Bcl-2) and reduced expression of pro-apoptotic proteins, including Bcl-2-associated X protein (BAX), caspase-3, and caspase-9. Notably, the autophagy inhibitor 3-methyladenine (3-MA) completely reverses palmatine’s protective effects, confirming its dependence on AMPK/mTOR pathway activation (Lin et al., 2022). In the context of non-alcoholic steatohepatitis (NASH), network pharmacology and molecular docking studies have revealed that the traditional Chinese medicinal compound formulations Ganlu Powder may treat NASH by modulating inflammatory responses and regulating phosphatidylinositol 3-kinase (PI3K) signaling. As a key active metabolite of Ganlu Powder, palmatine stably binds to protein kinase B Alpha (AKT1) with higher affinity than AKT1 inhibitors (Gao et al., 2022). This dual mechanism, enhancing autophagic clearance of damaged organelles via the AMPK/mTOR pathway while regulating inflammation and metabolic homeostasis through the AKT pathway, positions palmatine as a promising multi-target therapeutic agent for intervening in the progression of fatty liver diseases.

##### 4.1.2.2 Hepatic fibrosis

Palmatine exerts therapeutic effects by mitigating hepatic steatosis and suppressing inflammatory cytokine release. Molecular docking studies demonstrate its binding affinity for key fibrogenic proteins, including CYP1A1, ornithine decarboxylase 1 (ODC1), and monoamine oxidase B (MAOB) (Wang Q. et al., 2022). Experimental studies using a CCl_4_ induced liver fibrosis model in Sprague-Dawley rats demonstrated that palmatine administration at both low (54 mg/kg) and high (108 mg/kg) doses significantly reduced serum levels of ALT, AST, ALP, and GLB compared to the control group. Furthermore, palmatine treatment markedly decreased collagen deposition and improved pathological manifestations of liver fibrosis (Qin et al., 2023). The derivative compound 3a (IC_50_ = 8.19 μmol/L; selectivity index [SI] = 8.59) exhibited dose-dependent anti-fibrotic effects in a choline-deficient, L-amino acid-defined high-fat diet (CDAHFD)-induced NASH mouse model. Treatment significantly attenuated hepatic steatosis and inflammation, reduced collagen deposition-related proteins (COL1A1) protein deposition in liver tissue, and suppressed expression of pro-fibrotic factors including transforming growth factor-beta 1 (TGF-β1), α-smooth muscle actin (α-SMA) and tissue inhibitor of metalloproteinases 1 (TIMP1) (Zhang et al., 2023).

Metabolically, palmatine rectifies gut-liver axis dysfunction through a tripartite mechanism. First, based on proton nuclear magnetic resonance (1H-NMR) metabolomics analysis, it corrects metabolic abnormalities of gut microbiota-derived metabolites (including isoleucine and taurine) associated with hepatic fibrosis. Second, it modulates gut microbiota composition by specifically reducing the abundance of *Lactobacillus* species such as L. murinus, L. reuteri, and L. johnsonii. Third, it significantly enhances the production of short-chain fatty acids (SCFAs), particularly butyrate and propionate, which collectively contribute to improved intestinal barrier function and reduced hepatic inflammatory factor levels (Qin et al., 2023). This multi-target “anti-fibrotic–metabolic modulation–microbial homeostasis” paradigm presents a novel therapeutic strategy for hepatic fibrosis intervention.

Collectively, palmatine alleviates digestive disorders through multitargeted regulation of intestinal inflammation, gastric mucosal repair, and gut-liver axis metabolic homeostasis (Table 1). Its therapeutic effects primarily stem from anti-inflammatory, antioxidant, and mucosa-protective mechanisms. Given the anatomical and physiological features of the gastrointestinal system, palmatine’s molecular mechanisms primarily restore intestinal barrier integrity through multiple interconnected actions: inhibition of IDO-1-mediated tryptophan metabolic dysregulation, upregulation of tight junction protein ZO-1 expression, suppression of NLRP3 inflammasome activation and STAT1/CXCL10 signaling pathways, modulation of gut microbiota homeostasis, and activation of PINK1/Parkin-dependent autophagy. Regarding hepatoprotective effects, palmatine demonstrates comprehensive multi-target therapeutic potential for digestive system disorders—including colitis, gastritis, and hepatic fibrosis—through dual mechanisms of activating the AMPK/mTOR autophagy pathway to clear damaged organelles while simultaneously suppressing TGF-β1/α-SMA fibrotic signaling.

**TABLE 1:** Molecular mechanisms of palmatine in digestive system diseases.

Disease model	Study type	Experimental subjects/induction methods	Dose Range/Du ration	Key biological pathways	Molecular and cellular mechanisms of action	References
Colitis	*In vivo*	BALB/c mice, 3% DSS	50 mg/kg; 7 days	Nrf2; NLRP3 inflammasome	Nrf2↑, MPO↓, NLRP3↓	Cheng et al. (2022a)
	*In vivo*	SD rat, 5% DSS	25, 50, 100 mg/kg/d; 7 days	m6A methylation; Tight junction	METTL3, METTL14↑; ALKBH5, FTO↓; m6A methylation↑; ZO-1↑	Ji et al. (2024)
	*In vitro*	NCM460, 2% DSS	50, 100, 200 μg/mL; 24 h			
	*In vivo*	BALB/c mice, 3% DSS	50, 100 mg/kg; 7 days	Tryptophan metabolism	IDO-1↓	Zhang et al. (2018b)
	*In vivo*	BALB/c mice, 3% DSS	40, 100 mg/kg; 7 days	PINK1/Parkin-mediated mitophagy	PINK1, Parkin, LC3↑; NLRP3, IL-1β↓	Mai et al. (2019)
	*In vitro*	THP-Ms, PMA + LPS + ATP	300 μM; 3 h			
	*In vitro*	CD4^+^ T cells, CD3/CD28 Dynabeads	15.6, 31.25, 62.5, 125, 250, 500 μg/mL; 72 h	Treg differentiation	OXPHOS of DCs↑; Treg differentiation↑	Huang et al. (2024)
	*In vivo*	C57BL/6 mice, TNBS	1.94, 3.88, 7.76 g/kg; 11 days			
	*In vivo*	Rag1^−/−^ mice, T cells migration/TNBS	1.94, 3.88, 7.76 g/kg; 5 weeks			
CAG	*In vivo*	SD rat, *H. pylori*	10, 20, 40 mg/kg/d; 4 weeks	Mucosal barrier protein; Taurine/glycerophospholipid metabolism	IFN-γ↓; PG I, PG II, G-17, PG I/PG II↑	Chen et al. (2020b)
	*In vitro*	GES-1 cells *H. pylori*	10 μM, 20 μM, 40 μM; 12 h	ADAM17/EGFR signaling; MMP-mediated ECM remodeling	ADAM17/EGFR↓; MMP-10↓; CXCL16↓, Reg3a↑	Chen et al. (2020a)
	*In vivo*	SD rat, *H. pylori*	10, 20, 40 mg/kg/d; 4 weeks			
CAG	*In vivo*	C57BL/6J mice, MNNG (170 μg/mL)	45, 90, 180 mg/kg; 27 weeks	STAT1/CXCL10 signaling	PGA, PGC↓, GAST↑; H^+^/K^+^ ATPase↑; F4/80↓; STAT1, p-STAT1Y701↓; CXCL10, IL-17, TNF-α, p-p65↓; ROS↓	Zhou et al. (2024)
	*In vitro*	GES-1 cells, MNNG (20 µM)	20, 40, 80 μM; 12 h			
Hepatic Fibrosis	*In vivo*	SD rat, CCl_4_	54, 108 mg/kg; 4 weeks	XOD/urate transport; NLRP3 inflammasome	ALT, AST, ALP, GLB↓	Qin et al. (2023)
Alcoholic liver disease	*In vitro*	Mice hepatocyte, ethyl alcohol (100 Mm)	150 μg/mL; 24 h	AMPK/mTOR	AMPK/mTOR↑; LDH↓; LC3-II, ATG5, ATG7↑, p62↓; BAX, caspase-3, caspase-9↓, Bcl-2↑	Lin et al. (2022)

DSS, dextran sulfate sodium; Nrf2, Nuclear factor erythroid 2-related factor 2; MPO, myeloperoxidase; NLRP3, nod-like receptor protein 3; SD, Sprague-Dawley; METTL, methyltransferase-like; ALKBH, AlkB homolog; FTO, fat mass and obesity-associated protein; ZO-1, zonulin-1; NCM460, Normal Colonic Mucosa 460; IDO-1, indoleamine 2,3-dioxygenase-1; PINK1, PTEN-induced Putative Kinase 1; LC3, Light Chain 3; IL, interleukin; THP-Ms, THP-1-derived macrophages; PMA, Phorbol 12-myristate 13-acetate; LPS, lipopolysaccharide; ATP, adenosine triphosphate; CD, cluster of differentiation; OXPHOS, oxidative phosphorylation; CAG, chronic atrophic gastritis; DCs, Dendritic Cells; TNBS, 2,4,6-Trinitrobenzene Sulfonic Acid; IFN-γ, interferon-γ; PG, pepsinogen; G-17, Gastrin-17; GES-1, gastric epithelial cells; ADAM17, A Disintegrin And Metalloprotease 17; EGFR, epidermal growth factor receptor; MMP, matrix metalloproteinase; CXCL, C-X-C motif chemokine ligand; Reg3α, Regenerating Islet-derived Protein 3-alpha; MNNG, N-Methyl-N′-Nitro-N-Nitrosoguanidine; PGA, Pepsinogen A; PGC, Pepsinogen C; GAST, gastrin; H^+^/K^+^; ATPase, Hydrogen/Potassium Adenosine Triphosphatase; STAT, signal transducer and activator of transcription; p-STAT1Y701; TNF, tumor necrosis factor; ROS, oxygen species; GES, gastric epithelial cells; CCl_4_, carbon tetrachloride; ALT, alanine aminotransferase; AST, aspartate aminotransferase; ALP, alkaline phosphatase; GLB, globulin; AMPK, AMP-activated protein kinase; mTOR, mechanistic target of rapamycin; LDH, lactate dehydrogenase; ATG, autophagy-related genes; BAX, B-cell lymphoma 2 (Bcl-2)-associated x protein; caspase, Cysteine-dependent Aspartate-specific Protease; Bcl-2, B-cell lymphoma 2.

However, despite palmatine’s demonstrated multi-target intervention potential in digestive system disorders, significant knowledge gaps remain regarding its precise mechanisms of action. Particularly, the dynamic regulatory mechanisms within the “gut microbiota-intestinal barrier-liver function” interaction network have not been fully elucidated. With advancing research on gut microbiota, future studies should further investigate palmatine’s effects on microbial composition, metabolic products (such as short-chain fatty acids), and downstream signaling pathways, with special emphasis on its bidirectional regulatory role in the gut-liver axis.

### 4.2 Neurological and psychiatric disorders

#### 4.2.1 Neurodegenerative diseases

##### 4.2.1.1 Alzheimer’s disease

As a multi-pharmacological isoquinoline alkaloid, palmatine serves as a key active metabolite in traditional Chinese medicine extracts for Alzheimer’s disease (AD) treatment (Wang S. et al., 2022). It exerts anti-AD effects through synergistic modulation of critical pathological processes including the cholinergic system, mitochondrial homeostasis, oxidative stress and neuroinflammation.

Regarding cholinergic regulation, isoquinoline alkaloid extracts demonstrate acetylcholinesterase (AChE) inhibitory activity (Kaufmann et al., 2016). Palmatine exhibits particularly notable AChE inhibition (IC_50_ = 36.6 μM), surpassing other Coptis rhizoma alkaloids such as berberine, coptisine in potency (Zhao et al., 2016), and molecular docking studies also confirm its strong binding affinity with AChE (Zeng J. et al., 2024). Research further reveals that combinatorial administration of alkaloids, including berberine, coptisine, and palmatine, produces synergistic enhancement of acetylcholine inhibition (Kaufmann et al., 2016). This inhibitory effect effectively delays acetylcholine degradation and improves neurotransmitter balance. Meanwhile, label-free proteomic analysis revealed that the majority of differentially expressed proteins in the palmatine-treated group showed opposite trends compared to both the 5xFAD mouse control group and the normal blank group. These changes were particularly pronounced in the cerebellum and hippocampus, while exerting minimal impact on the cerebral cortex (Kiris et al., 2023). Multiple reaction monitoring (MRM) confirms palmatine’s blood-brain barrier permeability (Kiris et al., 2023), enabling direct central nervous system action to enhance cognitive function (Kiris et al., 2023; Pei et al., 2022) and ensuring pharmacological efficacy.

Notably, palmatine demonstrates unique mitochondrial protective properties. Natural alkaloid palmatine induces autophagy across multiple human cell lines (Lee et al., 2023). In the Aβ_1-40_-induced SD rat model with bilateral hippocampal injection, palmatine demonstrated superior neuroprotective effects compared to both the model group and the positive control (huperzine A) treatment group. The metabolite exerted its anti-AD effects by upregulating AMPK, Beclin-1, and LC3 expression while suppressing mTOR and P62 levels, thereby modulating the AMPK/mTOR autophagy signaling pathway and ameliorating neurological dysfunction (Han et al., 2024). Interestingly, studies have revealed that palmatine induces mitophagy without compromising mitochondrial function (Lee et al., 2023; Zhao et al., 2023). In PS2APP AD model mice, palmatine administration reversed the decline in mitochondrial membrane potential and restored both the time spent in the target quadrant and travel distance to levels comparable to wild-type mice. Further investigation demonstrated that this effect was associated with palmatine-mediated upregulation of the PGC-1α/Nuclear Respiratory Factor 1 (NRF1)/Mitochondrial Transcription Factor A (TFAM) pathway and reduction in pro-inflammatory cytokines IL-1β, IL-6, and TNF-α expression. These findings were further validated in PINK1 knockout models (Lee et al., 2023), suggesting that palmatine significantly ameliorates AD-related mitochondrial dysfunction and spatial learning/memory impairments. Furthermore, compared to clinical mitophagy inducers such as carbonyl cyanide m-chlorophenyl hydrazone (CCCP) or trifluoromethoxy carbonylcyanide phenylhydrazone (FCCP), the low toxicity of palmatine, manifested as absent membrane potential depolarization or reactive oxygen species (ROS) surge, provides superior therapeutic safety (Lee et al., 2023).

In AD treatment, palmatine exerts core antioxidant effects through activation of the Nrf2/HO-1 signaling pathway, constituting a critical link of its neuroprotective activity. Palmatine (50 mg/kg, 100 mg/kg) significantly improves neuronal survival rates in Aβ_25-35_-induced AD models using PC12 cells and mouse hippocampal neurons. The molecular mechanisms involve upregulation of Nrf2 and HO-1 expression while suppressing Kelch-like ECH-associated protein 1 (Keap-1) and BAX expression (Wang et al., 2023). This Nrf2/HO-1 pathway activation provides dual benefits: on one hand, it regulates ARE elements to promote antioxidant enzyme generation and reduce oxidative stress markers, thereby enhancing cellular antioxidant capacity. On the other hand, it inhibits the BAX/Bcl-2 pathway to decrease neuronal apoptosis (Jia et al., 2021; Wang et al., 2023).

Notably, palmatine’s anti-inflammatory effects synergize with its antioxidant pathway. Studies demonstrated that palmatine at 0.05 mg/mL showed no significant effect on Raw264.7 cell viability, while dose-dependently (0.025–0.5 mg/mL) downregulated LPS-induced IL-6 and IL-1β secretion (Pei et al., 2022). In APP/PS1 mouse models, the combined administration of palmatine and berberine resulted in more pronounced reductions in microglia-derived inflammatory factors (TNF-α, IL-1β) compared to monotherapy with either metabolite alone. This combination therapy simultaneously inhibited Aβ plaque formation and microglial activation while promoting neuronal repair and restoration of learning and memory functions (Zhao et al., 2023). This “antioxidant-anti-inflammatory-neuroprotective” triple-action network makes palmatine an effective natural metabolite for intervening in AD’s key pathological aspects of oxidative stress and neuroinflammation. Particularly noteworthy are recent findings that microglia-derived exosome (Exos)-encapsulated berberine/palmatine (Ber/Pal) delivery systems can improve drug blood-brain barrier penetration efficiency, enhance targeted accumulation in lesion areas, and further achieve multi-target intervention through regulation of the neuroinflammatory microenvironment (Zhao et al., 2023). This provides new strategies for developing targeted AD therapies based on natural metabolites and exosomes. This strategy not only enhances therapeutic efficacy through synergistic effects of natural metabolites, but also provides a novel approach for developing targeted AD therapies based on natural metabolites and exosomes. It represents a promising direction for improving blood-brain barrier targeting of natural medicines in future research.

##### 4.2.1.2 Parkinson’s disease

Parkinson’s disease (PD) is pathologically characterized by progressive loss of dopaminergic neurons in the substantia nigra and the presence of α-synuclein-immunoreactive inclusions (Lin C. H. et al, 2024). Studies show that palmatine exerts neuroprotective effects through multiple pathways. In 1-methyl-4-phenyl1,2,3, 6-tetrahydropyridine (MPTP)-induced Parkinson’s disease animal models, *Coptis chinensis Franch.* extract (containing palmatine) dose-dependently improved motor dysfunction and significantly increased the number of tyrosine hydroxylase-positive neurons in the substantia nigra (Friedemann et al., 2016). This neuroprotective effect has been clinically validated through a case-control study involving 1,016 Taiwanese PD patients and 539 healthy controls. The results demonstrated that β-glucocerebrosidase gene (GBA) L444P carriers exhibited a 3.93-fold increased risk of developing PD compared to normal controls. Palmatine exerts its protective effects on dopaminergic SH-SY5Y cells by upregulating GBA expression and activating autophagy, thereby reducing α-synuclein aggregation and associated neurotoxicity (Lin C. H. et al, 2024).

As a neurodegenerative disease, inflammation and mitochondrial dysfunction play key roles in the etiology and pathogenesis of Parkinson’s disease. At the molecular level, palmatine intervenes in key pathological processes of PD through dual pathways. On one hand, palmatine inhibits microglial activation, blocks NLRP3 inflammasome assembly and the release of other pro-inflammatory factors such as IL-6, TNF-α and NO, thereby alleviating neuroinflammation (Zhao et al., 2025). On the other hand, MPTP-induced Parkinson’s disease model mice exhibited significant reductions in LC3-II, autophagic substrate P62, and mitochondrial marker protein PINK1 levels. Palmatine treatment promoted the conversion of LC3-I to LC3-II while simultaneously inhibiting the degradation of LC3-II, autophagic substrate P62, and PINK1, thereby further enhancing mitophagy (Zhao et al., 2025). This regulation of mitochondrial quality control system can not only clear dysfunctional mitochondria but also maintain energy homeostasis in dopaminergic neurons, ultimately improving motor deficits and preventing dopaminergic neuronal damage.

##### 4.2.1.3 Ischemic stroke

The pathological progression of ischemic stroke involves a vicious cycle of oxidative stress and neuroinflammation, leading to irreversible neuronal damage. In the permanent focal cerebral ischemia mouse model induced by permanent middle cerebral artery occlusion (pMCAO), palmatine (0.2, 2 and 20 mg/kg) treatment significantly reduced infarct area and improved neurological deficits while preventing impairments in working and aversive memory. By blocking NF-κB nuclear translocation, it decreased expression of microglial and glial fibrillary acidic protein (GFAP) (astrocyte activation markers) and downregulated pro-inflammatory factors including TNF-α, IL-1β, inducible nitric oxide synthase (iNOS) and cyclooxygenase-2 (COX-2) (Pereira et al., 2023). Additionally, activation of the AMPK/Nrf2 pathway represents another crucial neuroprotective mechanism of palmatine. In the mouse model of transient middle cerebral artery occlusion (MCAO), oral administration of palmatine (50 mg/kg and 100 mg/kg) significantly enhanced AMPK phosphorylation and facilitated nuclear accumulation of Nrf2. Experimental evidence confirmed that Nrf2 gene silencing completely abolished these neuroprotective effects (Tang C. et al., 2021). Through dual mechanisms, NF-κB inhibition to alleviate neuroinflammation and AMPK/Nrf2 activation to enhance endogenous antioxidant defenses, palmatine exerts dose-dependent therapeutic effects in ameliorating cerebral edema, reducing infarct volume, and promoting neurological functional recovery.

#### 4.2.2 Pain and neural hyperexcitability

##### 4.2.2.1 Trigeminal neuralgia

The pathological process of trigeminal neuralgia (TN) involves abnormal purinergic signaling and dysregulation of neurotrophic factors. Regarding purinergic signaling modulation, palmatine (0.02 mL/100 g) significantly suppressed P2X7 receptor expression in the trigeminal ganglion (TG) of chronic constriction injury of the infraorbital nerve (CCI-ION)-induced TN rat models. The treatment concurrently inhibited p38 phosphorylation and reduced pro-inflammatory cytokine (IL-1β and TNF-α) production, effectively reversing mechanical allodynia and elevating the mechanical pain threshold. These effects effectively reverse mechanical allodynia and elevate mechanical pain thresholds by blocking P2X7 receptor-mediated aberrant neuron-glia pain transmission (Yin et al., 2021). In terms of neurotrophic factor regulation, palmatine reverses TN-induced hyperactivation of the brain-derived neurotrophic factor (BDNF)/tropomyosin receptor kinase B (TrkB) pathway and suppresses downstream extracellular signal-regulated kinase 1/2 (ERK1/2) phosphorylation, thereby interrupting the pain transmission in the TN rat model with loose ligation of the right infraorbital nerve (Liu L. et al, 2020). This dual mechanism - inhibiting pain perception through both purinergic pathways and neurotrophic factor modulation - provides the molecular basis for palmatine’s potential as a therapeutic agent for TN.

##### 4.2.2.2 Epilepsy

Palmatine demonstrates broad-spectrum anticonvulsant activity across multiple epilepsy models. In the model of epileptic seizures induced by timed infusion of pentantetrazole (PTZ) with palmatine in zebrafish larvae, palmatine (37.5, 75, 150, 300, 450 µM) dose-dependently suppressed epileptiform discharges (ED_50_ = 181.2 μM, 95% CI: 141.6–231.7 μM) and reduced PTZ-induced hyperkinetic behavior (Gawel et al., 2020). Furthermore, in ethyl 2-ketopent-4-enoate (EKP)-induced zebrafish epilepsy models or drug-resistant seizure models, palmatine decreased seizure frequency through potential modulation of glutamate decarboxylase activity and non-competitive antagonism of AMPA receptors (Nieoczym et al., 2024). Notably, unlike berberine’s GABA_A_ receptor-dependent inhibitory mechanism, palmatine’s antiepileptic effects occur without altering cerebral gamma-aminobutyric acid (GABA) levels. Instead, it exerts antiepileptic activity by significantly downregulating the epilepsy-associated immediate-early gene FBJ murine osteosarcoma viral oncogene homolog (c-Fos) expression and reducing neurotrophic factor BDNF levels - a gold-standard biomarker reflecting abnormal neuronal discharges and synaptic remodeling (Gawel et al., 2020). This mechanistic complementarity endows palmatine and berberine combination therapy with synergistic potential, offering novel combinatorial strategies for drug-resistant epilepsy.

#### 4.2.3 Mental and sleep disorders

##### 4.2.3.1 Depression

Palmatine exerts antidepressant effects through modulation of the monoaminergic system, oxidative stress, neuroinflammation, and apoptotic pathways. Behaviorally, Dhingra D et al. demonstrated that palmatine exhibits antidepressant-like activity comparable to fluoxetine in young male Swiss albino mice including non-stressed and stressed models. This effect may be achieved through inhibition of monoamine oxidase A (MAO-A) activity, reduction of plasma nitrite levels, and antioxidant activity. Furthermore, palmatine significantly reversed stress-induced increases in cerebral oxidative stress markers, catalase, lipid peroxidation, as well as corticosterone levels (Dhingra and Bhankher, 2014).

Regarding neuroinflammatory regulation, palmatine (5 mg/kg and 20 mg/kg) reduces levels of pro-inflammatory cytokines in LPS-induced mice and BV2 cells, while decreasing expression of CD68, iNOS, and CD206 proteins. Through the phosphodiesterase type 4B (PDE4B)/krüppellike factor 4 (KLF4) signaling pathway, it inhibits M1 microglial polarization and promotes M2 polarization, thereby alleviating depressive-like behaviors (Wang L. et al., 2022). Notably, in diabetic neuropathic pain (DNP) with comorbid depression (DP) rat models, palmatine treatment reduces hippocampal ERK1/2 phosphorylation levels and decreases expression of P2X7 receptors, astrocyte marker GFAP, and inflammatory factors, simultaneously improving hyperalgesia, allodynia, and depressive behaviors (Shen et al., 2018).

Network pharmacology studies further reveal that as a key active metabolite of Jiaotai Pill, palmatine synergizes with berberine, quercetin, and other metabolites to exert hypoglycemic and antidepressant effects, significantly improving depressive-like behaviors in CRS-induced db/db mice (Tang Y. et al., 2021). Its neuroprotective effects are also manifested through reduction of hippocampal neuronal apoptosis via the BAX/B-cell lymphoma-extra Large (Bcl-2) pathway and enhancement of antioxidant defenses via Nrf2/HO-1 pathway activation (Pei et al., 2023), establishing a multi-target antidepressant network.

##### 4.2.3.2 Insomnia

Palmatine exerts sedative-hypnotic effects through specific modulation of the serotonin system, demonstrating a clear dose-response relationship. In pentobarbital-induced sedative-hypnotic mouse models, oral administration of palmatine (119, 238, and 476 mg/kg) showed no direct soporific effects at any dose in autonomic activity tests. However, it significantly prolonged sleep duration and reduced sleep latency in suprathreshold pentobarbital-treated mice, while increasing sleep episodes and sleep incidence in subthreshold-dose groups. Additionally, palmatine treatment markedly suppressed spontaneous locomotor activity (Du et al., 2019). Neurochemical analyses indicate these effects may be mediated by palmatine’s ability to elevate cerebral 5-Hydroxytryptamine (5-HT) content without significantly affecting GABA levels. *In vitro* studies confirm that palmatine maintains PC12 cell viability within its effective concentration range (0.1–10 μM) (Du et al., 2019), demonstrating both sedative efficacy and safety advantages. These properties position palmatine as a distinctive candidate molecule for developing novel hypnotic agents.

Collectively, palmatine demonstrates distinctive central nervous system-targeting advantages in treating neuropsychiatric disorders. Its neuroprotective effects are mediated through multiple signaling pathways, including modulation of the cholinergic system, attenuation of oxidative stress, suppression of neuroinflammation, and enhancement of autophagy (Table 2). In neurodegenerative conditions, palmatine activates the TFEB/PINK1 signaling axis to potentiate AMPK/mTOR-mediated autophagy, thereby inducing mitophagy and significantly inhibiting the degradation of LC3-II, SQSTM1/p62, and PINK1. Furthermore, palmatine augments antioxidant defenses via the AMPK/Nrf2 and Nrf2/HO-1 pathways while blocking NLRP3 inflammasome assembly and suppressing NF-κB and iNOS/NO signaling cascades, ultimately reducing neuroinflammatory cytokine release and ameliorating psychiatric disturbances. For pain regulation, palmatine attenuates chronic nociception through dual inhibition of P2X7 receptor-mediated glial activation and aberrant BDNF/TrkB signaling hyperexcitability. In managing mental disorders, it modulates the PDE4B/KLF4 axis to promote microglial polarization toward the M2 phenotype and restores 5-hydroxytryptamine system homeostasis, revealing therapeutic potential against depression and anxiety. Notably, palmatine exhibits exceptional blood-brain barrier penetration. The advancement of exosome-based delivery systems may augment its bioavailability, thereby enhancing therapeutic efficacy in neuropsychiatric disorders—particularly its promise for neurodegenerative conditions such as Alzheimer’s and Parkinson’s diseases.

**TABLE 2:** Molecular mechanisms of palmatine in neurological and psychiatric disorders.

Disease model	Study type	Experimental subjects and induction methods	Dose Range/Du ration	Key biological pathways	Molecular and cellular mechanisms of action	References
AD	*In vivo*	PS2APP gene mutation mice	10 mg/kg; 4 weeks	Mitophagy	PGC-1α, NRF1, TFAM↑; IL-1β、IL-6, TNF-α↓; mtROS↓; COX-2, SDHB, MFN2↓; ATP↑	Lee et al. (2023)
	*In vitro*	BEAS-2B, A549, HeLa-Parkin, MEF cells	400 μM; 24 h			
	*In vivo*	APP/PS1 Double transgenic mouse	1.5 mg/kg; 21 days	NF-κB; Exosome-mediated targeting	PSD95↑; Aβ_40_, Aβ_42_, IBA-1↓; NO, TNF-α, IL-1β↓; IL-4, IL-10↑; Aβ plaque formation, Microglial quantity↓	Zhao et al. (2023)
	*In vitro*	Microglia, Aβ_25-35_ (20 μM)	1 μM; 24 h			
	*In vivo*	Kunming mice, LPS	100, 50 mg/kg; 7 days	PI3K/AKT	PI3K, p-AKT↓; IL-6, IL-1β, TNF-α↓	Pei et al. (2022)
	*In vitro*	Raw264.7, LPS	0.025, 0.05, 0.1, 0.2, 0.3, 0.4, 0.5 mg/mL; 48 h			
	*In vivo*	5xFAD transgenic mice	5, 10 mg/kg; 7 days	Oxidative stress; Ribosomal protein	HS105, HS12A, RL12↑	Kiris et al. (2023)
	*In vivo*	ICR mice, Aβ_25-35_ (2 μg/μL)	50, 100 mg/kg; 29 days	Nrf2/HO-1	Nrf2/HO-1↑; Bcl-2↑; Keap-1, BAX↓	Wang et al. (2023)
	*In vitro*	PC12 cells	0.1, 0.2, 0.3, 0.4, 0.5 mg/mL; 12 h		TNF-α, IL-1β, IL-6↓; MDA, ROS↓; GSH, SOD↑	
	*In vivo*	SD rat, Aβ_1-40_	280 mg/kg; 4 weeks	AMPK/mTOR; Gut microbiota-SCFAs	AChE↓; ACh, ChAT↑; Tau, Aβ↓; AMPK↑, mTOR↓; LC3, Beclin-1, P62↑; Neuronal apoptosis↓	Han et al. (2024)
	*In vitro*	HT22 cells, Aβ_1-40_	0.025, 0.05, 0.1, 0.2, 0.4, 0.6, 0.8, 1 mg/mL; 24 h		Cell apoptosis↓	
	*In vivo*	C57BL/6J mice, Aβ_1-42_	1.60 mg/kg, 16d	NF-κB; p38 MAPK; Exosome-mediated targeting	NF-κB, p38 MAPK↓; TNF-α, IL-1β↓; IL-4, IL-10↑; Aβ plaque formation↓	Zhou et al. (2025)
	*In vitro*	BV2 cells, LPS (166 µM)	1 μM, 24 h			
PD	*In vivo*	C57BL/6 mice, MPTP	50, 100, 200 mg/kg; 7 days	PINK1/Parkin-mediated mitophagy; NLRP3 inflammasome	NF-κB, NLRP3, caspase-1, IL-1β↓; LC3-II, P62, PINK1↑	Zhao et al. (2025)
	*In vitro*	Primary neuron and BV2 cells, MPP + LPS	5, 10, 20 μmol/L; 2 h			
IS	*In vivo*	Swiss mice, surgery	0.2, 2, 20 mg/kg; 4 days	Neuroinflammation-related; NF-κB; iNOS/NO	TNF-α, iNOS, COX-2, NF-κB↓; Activation of microglia and astrocytes↓	Pereira et al. (2023)
	*In vivo*	C57BL/6J mice, surgery	50, 100 mg/kg; 24 h	AMPK/Nrf2	AMPK/Nrf2↑, Neuronal apoptosis↓	Tang et al. (2021a)
	*In vitro*	PC12 cells, H/R	100, 200 μM; 6 h			
TN	*In vivo*	SD rat, surgery CCI-ION	0.02 mL/100 g; 14 days	P2X7 receptor	P2X7↓; IL-1β, TNF-α, p-p38↓; p38 MAPK↓	Yin et al. (2021)
	*In vivo*	SD rat, surgery	20 mg/kg; 14 days	BDNF/TrkB	BDNF, TrkB↓; BDNF/TrkB↓, p-ERK1/2↓	Liu et al. (2020a)
Seizures	*In vivo*	6 dpf, EKP (400 µM)	75–450 μM; 24 h	Glutamatergic; GABAergic; GAD-mediated GABA synthesis	PV-IR neurons become smaller; Excitatory neurotransmission↓	Nieoczym et al. (2024)
	*In vivo*	Swiss mice, TEAS/PTZ	10–80 mg/kg; 6 weeks			
	*In vivo*	7 dpf, PTZ	37.5, 75, 150, 300, 450 μM; 24 h	c-Fos/CREB	c-Fos, BDNF↓	Gawel et al. (2020)
DP	*In vivo*	Swiss albino mice, CUMS	0.25, 0.5, 1 mg/kg; 21 days	Monoaminergic neurotransmission	MAO-A, LOP↓; NO, CS↓	Dhingra and Bhankher (2014)
	*In vivo*	ICR, LPS (0.83 mg/kg) + CUMS	5, 20 mg/kg; 7 days	PDE4B/KLF4	PDE4B↓; KLF4↑; TNF-α, IL-6↓, CD68, iNOS↓; IL-4, IL-10↑; CD206, Arg1, Ym1↓	Wang et al. (2022a)
	*In vitro*	BV2 cells, LPS (1 μg/mL)	4, 8, 16 μM; 2 h			
DP	*In vivo*	BALB/c mice, LPS (5 g/L, 3 μL/mice)	50, 100 mg/kg; 14 days	BAX/Bcl-2 apoptosis; Nrf2/HO-1	BAX↓, Bcl-2↑; Nrf2, HO-1↑; SOD↑; TNF-α, IL-1β, IL-6↓; Neuronal apoptosis↓	Pei et al. (2023)
	*In vitro*	HT-22 cells, LPS (1 μg/mL)	0.025, 0.05, 0.1, 0.2, 0.4, 0.8, 1 mg/mL; 12 h		ROS↓; Cell apoptosis↓	
DNP with DP	*In vivo*	SD rat, HFD + STZ (35 mg/kg) + CUS	30 mg/kg; 14 days	P2X7 receptor; ERK1/2	p-ERK1/2↓; P2X7 receptor↓; TNF-α, IL-1β, GFAP↓	Shen et al. (2018)
Insomnia	*In vivo*	KM mice, pentobarbital sodium	119, 238, 476 mg/kg; 30 days	Serotonin (5-HT)	5-HT↑	Du et al. (2019)
	*In vitro*	PC12 cells	1, 0.1, 0.01 μg; 48 h			

AD, Alzheimer’s disease; mtROS, mitochondrial reactive oxygen species; ATP, adenosine triphosphate; BEAS-2B, human bronchial epithelial cell line; A549, Adenocarcinomic Human Alveolar Basal Epithelial Cells; MEF, mouse embryonic fibroblast; PSD95, Postsynaptic Density Protein 95; Aβ,Amyloid-beta; IBA-1, Ionized Calcium-Binding Adapter Molecule 1; NO, nitric oxide; TNF, tumor necrosis factor; caspase, Cysteine-dependent Aspartate-specific Protease; IL, interleukin; NeuN, LPS, lipopolysaccharide; PI3K, Phosphoinositide 3-Kinase; AKT, protein kinase B; Nrf2, Nuclear factor erythroid 2-related factor 2; HO-1, heme oxygenase-1; Bcl-2, B-cell lymphoma 2; Keap-1, Kelch-like ECH-associated protein 1; BAX, B-cell lymphoma 2 (Bcl-2)-associated x protein; MDA, malondialdehyde; ROS, reactive oxygen species; GSH, glutathione; SOD, superoxide dismutase; SD, Sprague-Dawley; AChE, acetylcholinesterase; Ach, Acetylcholine; ChAT, choline acetyltransferase; Tau, tubulin associated unit; AMPK, AMP-activated protein kinase; mTOR, mechanistic target of rapamycin; LC3, Light Chain 3; PD, Parkinson’s disease; MPTP, 1-Methyl-4-Phenyl-1, 2,3,6-Tetrahydropyridine; MPP, 1-Methyl-4-phenylpyridinium; NF-κB, nuclear factor-κB; NLRP3, nod-like receptor protein 3; PINK1, Putative Kinase 1; IS, ischemic stroke; iNOS, inducible nitric oxide synthase; COX-2, Cyclooxygenase-2; TN, trigeminal neuralgia; H/R, Hypoxia/Reoxygenation; CCI-ION, chronic constriction injury of the infraorbital nerve; P2X7, Purinergic Receptor P2X 7; MAPK, Mitogen-Activated Protein Kinase; EKP, ethyl 2-ketopent-4-enoate; GABA, gamma-aminobutyric acid; GAD, glutamic acid decarboxylase; BDNF, brain-derived neurotrophic factor; TrkB, tropomyosin receptor kinase B; ERK1/2, extracellular signal-regulated kinase 1/2; 6 dpf, 6 Days Post-Fertilization; TEAS, transcutaneous electrical acupoint stimulation; PTZ, pentylenetetrazol; c-Fos, CUMS, chronic unpredictable mild stress; CREB, cAMP, Response Element-Binding Protein; MAO-A, Monoamine Oxidase A; LOP, lipid peroxidation; CS, corticosterone; DP, depression; PDE4B, Phosphodiesterase Type 4B; KLF4, Krüppellike factor4; CD, cluster of differentiation; Arg1, Arginase 1; Ym1, Chitinase 3-Like 3; HFD, High-Fat Diet; STZ, streptozotocin; GFAP, glial fibrillary acidic protein; 5-HT, 5-Hydroxytryptamine.

### 4.3 Metabolic diseases

#### 4.3.1 Disorders of glucose and lipid metabolism

##### 4.3.1.1 Diabetes mellitus

Diabetes mellitus, a metabolic disorder characterized by insulin resistance and β-cell dysfunction, is pathologically associated with oxidative stress and chronic low-grade inflammation. In pancreatic β-cell protection, palmatine significantly inhibits expression of endoplasmic reticulum stress markers glucose regulated protein 78kda (GRP78) and calreticulin (CALR) in streptozotocin (STZ)-induced diabetic models, while upregulating antioxidant proteins including peroxiredoxin 4 (Prdx4), protein disulfide isomerases (PDIA2/3), and glutathione S-transferases (GSTs) (Okechukwu et al., 2021). It reduces β-cell apoptosis by blocking the ERK/c-Jun N-terminal Kinase (JNK) signaling pathway and promotes glucagon-like peptide-1 (GLP-1) secretion and insulin release (Tian et al., 2020; Yang et al., 2024).

For improving insulin sensitivity in target tissues, network pharmacology demonstrates that palmatine activates the IRS1/AKT2/FOXO1/GLUT2 signaling axis, reducing hepatic glucose output by 45%. Through hydrophobic interactions and π-π stacking, it directly inhibits α-amylase (IC_50_ = 28.3 μM) and α-glucosidase (Binding energy = −9.2 kcal/mol), thereby delaying intestinal carbohydrate absorption and exerts hypoglycemic effects (Chen et al., 2021; Nwabueze et al., 2022; Xia et al., 2022). Notably, in type 2 diabetes mellitus models, palmatine shows better restorative effects on insulin signaling pathways compared to metformin and glimepiride, and reduces serum high mobility group box 1 (HMGB1) levels to promote healing of diabetic corneal ulcers (Li et al., 2022; Nwabueze et al., 2022). Additionally, studies suggest a bidirectional relationship between diabetes and depression, proposing that insulin resistance (IR) and related metabolic disorders may increase risk of major depressive disorder (MDD) (Sen et al., 2021), providing new directions for cross-disciplinary research.

##### 4.3.1.2 Hyperlipidemia

Palmatine exerts lipid-lowering effects through synergistic regulation of cholesterol synthesis, bile acid metabolism, and gut microbiota balance. In cholesterol metabolism, Palmatine (23.35, 46.70, and 70.05 mg/kg) significantly upregulated hepatic low-density lipoprotein receptor (LDLR) and cholesterol 7α-hydroxylase (CYP7A1) expression while suppressing 3-hydroxy-3-methylglutaryl-CoA reductase (HMGCR) expression in high-fat diet (HFD)-induced hyperlipidemic hamster models (Kou et al., 2016; Ning et al., 2015), resulting in decreased serum levels of total cholesterol (TC), TG, and low-density lipoprotein cholesterol (LDL-C) in hyperlipidemic hamsters, along with increased fecal excretion of TC and total bile acids (TBA) (Kou et al., 2016; Ning et al., 2015; Ning et al., 2020).

Regarding bile acid metabolism regulation, palmatine activates CYP7A1 through the PPARα-CYP7A1 pathway while suppressing FXR expression, thereby promoting cholesterol conversion to bile acids (Ning et al., 2020). This dual regulatory effect is accompanied by improved gut microbiota composition and reduced bile acid enterohepatic circulation through downregulation of apical sodium-dependent bile acid transporter (ASBT) (Ning et al., 2015; Ning et al., 2020). Both *in vitro* and *in vivo* studies have demonstrated that palmatine exhibits synergistic effects when combined with other alkaloids (such as berberine and coptisine), effectively suppressing body weight gain, reducing serum total cholesterol, and increasing high-density lipoprotein cholesterol (HDL-C) in hyperlipidemic hamster models (He et al., 2016). In HepG2 cell models, the alkaloid combination more effectively reduces lipid and cholesterol accumulation by cooperatively inhibiting HMGCR mRNA expression to delay cholesterol synthesis, while promoting increased expression of LDLR, CYP7A1, and uncoupling protein-2 (UCP-2) to accelerate lipid clearance (He et al., 2016).

This multi-target mode of action integrating cholesterol metabolism, bile acid transformation, and gut microbiota regulation not only explains palmatine’s lipid-lowering efficacy but also provides a theoretical basis for developing natural product-based combination lipid-lowering strategies.

#### 4.3.2 Purine and bone metabolism disorders

##### 4.3.2.1 Hyperuricemia and Osteoarthritis

**FIGURE 4:**
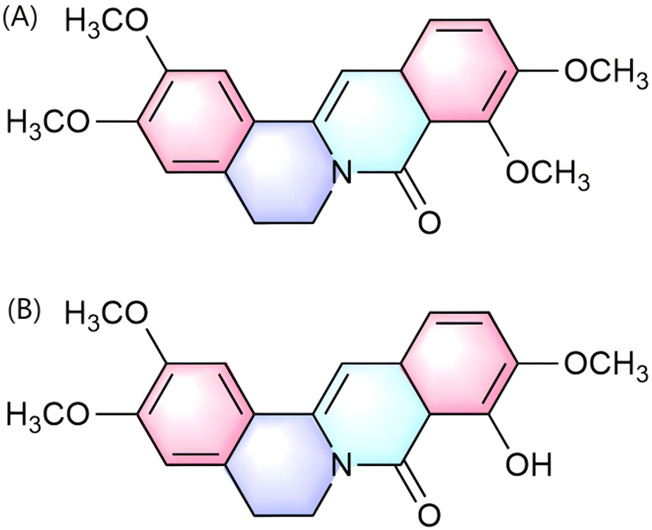
Chemical structure of 8-oxypalmatine **(A)** and 9-Hydroxy-8-oxypalmatine **(B)**.

Palmatine exerts uric acid-lowering effects through dual pathways: direct inhibition of xanthine oxidase (XOD) activity and modulation of renal urate transporters, thereby coordinately intervening in the pathological progression of hyperuricemia (HUA) and its complication gouty arthritis. Regarding uric acid metabolism regulation, the hepatic metabolite of palmatine, 9-Hydroxy-8-oxypalmatine (5, 10, and 20 mg/kg) (Figure 4B), significantly inhibited XOD and adenosine deaminase (ADA) activities in potassium oxonate- and hypoxanthine-induced HUA mouse models. It concurrently downregulated urate transporter 1 (URAT1) and glucose transporter 9 (GLUT9) while reversing the downregulation of organic anion transporter 1 (OAT1), thereby exerting anti-hyperuricemic and renal protective effects (Wu et al., 2024). Further investigations by Gaoxiang et al. revealed that palmatine treatment (25, 50, and 100 mg/kg) activated the Keap1-Nrf2 pathway to alleviate oxidative stress and suppressed TXNIP/NLRP3 inflammasome activation in HUA model mice, consequently ameliorating HUA-associated renal injury (Ai et al., 2023). Notably, palmatine’s primary metabolite 8-oxypalmatine (Figure 4A) also inhibits NLRP3 inflammasome assembly, suggesting its metabolite group may collectively contribute to anti-inflammatory and organoprotective effects (Cheng et al., 2022b).

In gouty arthritis models, both *in vitro* and *in vivo* studies demonstrated that palmatine (20, 40, and 80 μM) dose-dependently reduced LPS-induced expression of proinflammatory cytokines (IL-1β, IL-6, IL-18, and TNF-α) in THP-1 cells. In MSU-induced gouty arthritis mouse models, treatment with palmatine (25, 50, and 100 mg/kg) significantly attenuated joint inflammation and swelling while reducing neutrophil infiltration in synovial tissue. Mechanistically, palmatine inhibited the NF-κB/NLRP3 signaling pathway by suppressing phosphorylation of p65 and IκBα, while enhancing antioxidant protein expression (Nrf2 and HO-1) and elevating superoxide dismutase (SOD) and GSH levels, thereby providing comprehensive protection against MSU-induced inflammation and oxidative stress (Cheng et al., 2022a). From the perspective of inflammatory factor regulation, this effect is associated with palmatine’s inhibition of NLRP3 inflammasome activation and pyroptosis. It suppresses apoptosis-related proteins such as NLRP3, apoptosis-associated speck-like protein (ASC), caspase-1, IL-1β, HMGB1, and Cathepsin B, while reducing NETosis-associated proteins including caspase-11, histone H3, PR3, and PAD4. Moreover, it upregulates MMP-3 protein expression, and alleviates MSU-induced gouty arthritis inflammation (Jiang et al., 2025). Zhou X et al. demonstrated that intra-articular injection of palmatine (100 mg/L, administered weekly) significantly attenuated cartilage damage in rabbit osteoarthritis (OA) models induced by bilateral anterior cruciate ligament transection (ACLT). The chondroprotective effects were achieved through dual modulation of signaling pathways, specifically by inhibiting Wnt/β-catenin signaling while enhancing cyclopamine-mediated suppression of the Hedgehog pathway (Zhou et al., 2016).

As an important member of benzylisoquinoline alkaloids (palmatine 0.03%, tetrahydropalmatine 0.003%), palmatine synergistically inhibits calcium oxalate crystal nucleation and aggregation with other alkaloids, demonstrating significant anti-lithic activity. Through its antioxidant and anti-inflammatory effects, it exhibits pleiotropic benefits in the prevention and treatment of urolithiasis and gout (Misra et al., 2024). This multidimensional therapeutic network integrating “uric acid metabolism regulation-inflammasome inhibition-oxidative stress reduction - gut microbiota modulation” establishes palmatine as a potential multi-target drug for treating HUA and its articular complications.

##### 4.3.2.2 Osteoporosis

Palmatine ameliorates osteoporosis (OP) through dual pathways of “gut microbiota-bone metabolism” regulation and “osteoclast inhibition.” Oral administration of palmatine (100 mg/kg via gavage) significantly increased the abundance of gut microbiota including firmicutes, bacteroidota, and actinobacteria in OA-OP (osteoarthritis-osteoporosis comorbidity) model rats. Concurrently, it upregulated the levels of key metabolites such as 5-methoxytryptophan and β-tyrosine. These effects further enhance bone-protective markers like E2, ALP, BGP, 1,25(OH)_2_D_3_, reduce calcium loss, and effectively alleviate cartilage destruction and bone loss (Jie et al., 2023).

Regarding osteoclast suppression, palmatine dose-dependently (40 μM) induces osteoclast apoptosis via the nitric oxide synthase (NOS) system, significantly reducing osteoclast numbers in tissues while increasing NO metabolites (NO2-) and iNOS expression in NO-induced osteoclasts. This apoptotic effect is blocked by the pan-NOS inhibitor N(G)-nitro-L-arginine methyl ester hydrochloride, which inhibits iNOS (Ishikawa et al., 2015; Ishikawa et al., 2016). In Ovariectomy (OVX) mouse models, palmatine treatment (1 mg/kg and 10 mg/kg) significantly suppressed osteoclast numbers in bone tissues while inhibiting the receptor activator of nuclear factor-κB ligand (RANKL) signaling pathway. The metabolite blocked NF-κB activation and reduced serum levels of RANKL and osteoprotegerin (OPG), dose-dependently lowering the RANKL/OPG ratio (Ishikawa et al., 2015).

Metabolic disorders, including diabetes mellitus, hyperlipidemia, and hyperuricemia, involve complex pathogenesis driven by insulin resistance, oxidative stress, and chronic inflammation. As can be concluded above, palmatine, as a multitarget natural alkaloid, coordinates metabolic homeostasis through crosstalk among insulin signaling, oxidative stress, and purine metabolic pathways (Table 3). In glucolipid metabolism regulation, it enhances GLUT4 membrane translocation via the IRS1/AKT2/FOXO1 cascade while activating the PPARα-CYP7A1 axis to promote cholesterol-to-bile acid conversion. Concurrently, palmatine reduces endoplasmic reticulum stress, augments antioxidant enzyme activity, and suppresses NLRP3 inflammasome activation and inflammatory cytokine release, thereby restoring insulin sensitivity and lipid equilibrium. These integrated mechanisms underpin its broad-spectrum therapeutic potential against diabetes, hyperlipidemia, hyperuricemia, and osteoporosis.

**TABLE 3:** Molecular mechanisms of palmatine in metabolic diseases.

Disease model	Study type	Experimental subjects and induction methods	Dose Range/Du ration	Key biological pathways	Molecular and cellular mechanisms of action	References
DM	*In vivo*	SD rat, STZ (45 mg/kg)	2 mg/kg; 12 weeks	ER stress; Antioxidant enzyme	CALR, GRP78↓; Prdx4, PDIA2/3, GST, ALB↑; HbA1c, RBC↓; AST, ALT, AP↓; TG, TC, LDL-C↓; WBC↑; HDL-C↑; SOD, LPO, CAT, GSH↑	Okechukwu et al. (2021)
	*In vivo*	KK-Ay mice, HFD	225 mg/kg; 40 days	Lipid metabolism	TC, TG↓; HDL-c↑	Ma et al. (2016a)
	*In vitro*	HepG2 cells	0.2, 1, 5 mg/mL; 24 h			
T2DM	*In vivo*	Wistar rat and BALB/C mice, STZ (50 mg/kg)	12 mg/kg; 4 weeks	Glucose metabolism; Lipid metabolism; Oxidative stress	Blood glucose, HbA1c↓; TC, LDL, TG↓; ALP, AST, SGPT↓; HDL↑, MDA↓, SOD, Activation of CAT↑; Activation of α-amylase and α-glucosidase↓	Alamzeb et al. (2024)
	*In vivo*	SD rat, STZ (50 mg/kg)	5.85 µM; 8 weeks	IRS/PI3K/AKT/GLUT4; PKC-α	IRS, PI3K, IRS-1, PKC, AKT2↑, PKC-α↓, Tyrosine phosphorylation↑, GLUT4↑; JNK↓; β-cell apoptosis↓	Nwabueze et al. (2022)
	*In vitro*	L6 rat skeletal muscle cells, glucose (25 mM) + insulin (100 mM)	2 μM; 24 h			
IGT	*In vivo*	SD rat, HFD	40 mg/kg/day; 10 weeks	ERK/JNK	MAPK pathway↓; ERK, JNK, p-ERK, p-JNK↓; Cell apoptosis↓	Tian et al. (2020)
	*In vitro*	INS-1 cells, PA	0, 10, 20, 40 μg/mL; 48 h			
HLP	*In vivo*	Hamsters, HFD	23.35, 46.70, 70.05 mg/kg; 4 weeks	Cholesterol metabolism; Bile acid synthesis; LDLR regulatory; ASBT-mediated bile acid reabsorption	TC, TG, LDL-C↓; TBA↑; LDLR, CYP7A1↑, ASBT↓	Ning et al. (2015)
	*In vivo*	SD rat, HFD	23.35, 46.70, 70.05 mg/kg; 4 weeks	Bile acid metabolism (PPARα-CYP7A1)	TC, TG, LDL-C↓TBA↑; CYP7A1, PPARα↑; FXR↓; ZO-1, ZO-2, Claudin-1↑	Ning et al. (2020)
HUA	*In vivo*	Kunming mice, PO (300 mg/kg) + HX (300 mg/kg)	25, 50, 100 mg/kg; 1 week	Keap1/Nrf2; TXNIP/NLRP3	UA, CRE, BUN↓; Nrf2↑; TXNIP, NLRP3, ASC, caspase-1, IL-1β, IL-18↓; XOD, ADA↓	Ai et al. (2023)
GA	*In vivo*	Kunming mice, MSU	25, 50, 100 mg/kg	NF-κB; NLRP3 inflammasome	NF-κB, NLRP3↓; IL-1β, IL-6, IL-18, TNF-α, MDA↓; SOD, GSH↑; p-p65, p-IκBα↓; NF-κB, NLRP3, ASC, IL-1β, caspase-1↓; Nrf2, HO-1↑	Cheng et al. (2022a)
	*In vitro*	THP-1 cells, LPS (500 ng/mL)	20, 40, 80 μM; 1 h			
	*In vitro*	MPM cells, rat articular cartilage cells, LPS + MSU	6.25, 12.5, 25, 50, 100, 200 μM; 24 h	NLRP3 inflammasome; NETosis	NLRP3↓; caspase-1, caspase-11↓; IL-1β↓; NETs↓; pyroptosis↓; MMP-3↑	Jiang et al. (2025)
	*In vivo*	Kunming mice, MSU	25, 50, 100 mg/kg; 24 h/5 h			
Arthritis	*In vitro*	Rabbit knee cartilage cells, IL-1β	10, 25, 50, 100 mg/L; 24/48/72 h	Wnt/β-catenin; Hedgehog	Wnt/β-catenin↓; β-catenin↓; IL-1β↓; MMP-1, MMP-3, MMP-13↓; Hedgehog pathway↓, Ihh, Shh, Gli-2↓; DKK-1↑; TIMP-1, Collagenase II, aggrecan↑	Zhou et al. (2016)
	*In vivo*	New Zealand rabbits, ACLT	100 mg/L; 5 weeks			
OA and OP	*In vivo*	SD rat, ACLT + OVX	100 mg/kg; 56 days	Gut microbiota-host metabolic; Estrogen; Vitamin D; Tryptophan metabolism	E2, ALP, BGP, 1, 25(OH) 2D3↑; Ca↓	Jie et al. (2023)
OP	*In vitro*	RAW 264.7 cells, MC3T3-E1 cells	1, 5, 10, 40, 100 μM; 24 h-5 days	iNOS/NO	Cell apoptosis↑; Cell viability↓; NO↑, iNOS↑	Ishikawa et al. (2016)
	*In vivo*	ICR mice, OVX	1, 10 mg/kg, 13 weeks	RANKL/RANK/OPG	Osteoclast quantity↓; RANKL, OPG↓; RANKL/OPG ratio↓	Ishikawa et al. (2015)
	*In vitro*	MC3T3-E1 cells	10, 20, 40, 100, 200 μM; 24 h			

DM, diabetes mellitus; SD, Sprague-Dawley; STZ, streptozotocin; CALR, calreticulin; GRP78, Glucose Regulated Protein 78 kDa; Prdx4, Peroxiredoxin 4; PDIA2/3, Protein Disulfide Isomerase A2/A3; GST, Glutathione S-Transferase; ALB, albumin; T2DM, Type 2 Diabetes Mellitus; HbA1c, glycated hemoglobin; RBC, red blood cell; AST, aspartate aminotransferase; ALT, alanine aminotransferase; AP, alkaline phosphatase; TG, trigeminal ganglion; TC, total cholesterol; LDL-C, low-density lipoprotein cholesterol; WBC, white blood cell; HDL-C, High-Density Lipoprotein Cholesterol; SOD, superoxide dismutase; caspase, Cysteine-dependent Aspartate-specific Protease; LPO, lipid peroxidation; CAT, catalase; GSH, glutathione; HFD, High-Fat Diet; ALP, alkaline phosphatase; SGPT, Serum Glutamic-Pyruvic Transaminase; MDA, malondialdehyde; IRS, insulin receptor substrate; PI3K, phosphatidylinositol 3-kinase; PKC, Protein Kinase C; AKT, protein kinase B; GLUT4, Glucose Transporter 4; JNK, c-Jun N-terminal Kinase; IGT, impaired glucose tolerance; MAPK, Mitogen-Activated Protein Kinase; ERK, extracellular signal-regulated kinase; TBA, total bile acids; LDLR, Low-Density Lipoprotein Receptor; CYP7A1, Cholesterol 7α-Hydroxylase; ASBT, Apical Sodium-Dependent Bile Acid Transporter; PPARα, peroxisome proliferator-activated receptor Alpha; FXR, farnesoid X receptor; ZO, zonulin-1; HUA, hyperuricemia; PO, potassium oxonate; HX, hypoxanthine; UA, uric acid; CRE, creatinine; BUN, blood urea nitrogen; Nrf2, Nuclear Factor Erythroid 2-Related Factor 2; TXNIP, Thioredoxin-Interacting Protein; NLRP3, nod-like receptor protein 3; ASC, apoptosis-associated speck-like protein; IL, interleukin; XOD, xanthine oxidase; ADA, adenosine deaminase; GA, gastrin; MSU, monosodium urate; LPS, lipopolysaccharide; NF-κB, nuclear factor-κB; TNF, tumor necrosis factor; IκBα, Inhibitor of kappa B-α; HO-1, heme oxygenase-1; MPM, malignant pleural mesothelioma; NETs, Neuroendocrine Tumor cells; MMP, matrix metalloproteinase; Ihh, Indian Hedgehog; Shh, Sonic Hedgehog; Gli, Glioma-associated oncogene homolog; DKK-1, Dickkopf-related protein 1; TIMP-1, Tissue Inhibitor of MMP-1; ACLT, anterior cruciate ligament transection; OA, Osteoarthritis; OP, osteoporosis; OVX, ovariectomy; E2, estradiol; BGP, Osteocalcin (Bone Gla Protein); NO, nitric oxide; iNOS, inducible nitric oxide synthase; RANKL, Nuclear Factor-κB ligand receptor activator; OPG, osteoprotegerin.

### 4.4 Cancer

#### 4.4.1 Direct tumor cell killing effects

Palmatine directly inhibits tumor cell growth and induces programmed cell death through multiple mechanisms. In colorectal cancer, palmatine significantly prolongs the survival of Apc^Min/+^ mice and reduces small intestine and colon tumor numbers in a dose-dependent manner (Ma W. K. et al., 2016). The mechanism involves targeting Aurora kinase A (AURKA), inducing G2/M phase arrest in colon cancer cells, while regulating mitochondrial-related apoptotic pathways. This leads to decreased Bcl-xl and Bcl-2 protein levels, along with increased expression of pro-apoptotic proteins like P53, P73, caspase-3, caspase-9 and cytochrome c (cyt.c) (Liu X. et al, 2020). Network pharmacology and biological studies have shown that palmatine can regulate cell cycle regulatory-related proteins such as MMP, Bcl-2, E2F1and so on (Fan T. et al., 2022). Animal studies demonstrated that palmatine exerts anti-oral squamous cell carcinoma (OSCC) effects by inhibiting PI3K/AKT/mTOR signaling pathway activation, upregulating BAX expression, and suppressing Bcl-xl and Bcl-2 expression, thereby inducing autophagy and promoting apoptosis in CAL27 cells (29). *In vitro* experiments further confirmed that palmatine (5.5–7.9 µM) concentration-dependently induced apoptosis in OVCAR-4 cancer cells through enhanced caspase-3/7 activity and poly (ADP-ribose) polymerase cleavage, consequently inhibiting ovarian cancer cell growth (Johnson-Ajinwo et al., 2019). This concentration-dependent effect was also observed in the suppression of breast cancer cell viability and proliferation, while palmatine showed no significant impact on normal human mammary epithelial cell growth (Grabarska et al., 2021).

#### 4.4.2 Metastasis and invasion inhibition

Palmatine effectively inhibits tumor metastasis and prevents cell invasion. In triple-negative breast cancer lung metastasis models, palmatine treatment dose-dependently improves metastatic lung lesions by downregulating metastasis-associated protein 1 (MTA1) and upregulating tumor suppressor p53 expression, while preserving lung morphology (Ativui et al., 2022). In a murine model of triple-negative breast cancer with lung metastasis, palmatine (1, 5, and 10 mg/kg) demonstrated dose-dependent therapeutic efficacy by significantly ameliorating metastatic lung injury and hypoxemia, downregulating metastasis-associated protein 1 (MTA1), while upregulating the expression of the tumor suppressor p53, thereby preserving pulmonary histomorphology (Ativui et al., 2022). Furthermore, *in vitro* and *in vivo* studies utilizing the CMT cell line CMT-U27 and its xenograft mouse model revealed that palmatine administration (50 mg/kg *in vivo*; 50, 100, and 200 μM *in vitro*) suppressed primary tumor growth and adjacent lymph node metastasis in breast cancer models by downregulating the protein expression of PI3K, PTEN, and AKT, thereby inhibiting PI3K/AKT pathway activation (Yoo et al., 2023).

*In vitro* investigations demonstrated that human pancreatic cancer cell lines (HPNE, HPNE-Ras, MIA PaCa-2, PANC-1, and CFPaC-1) interact with pancreatic stellate cells (PSCs). Palmatine induced apoptosis by suppressing survivin and glioma-associated oncogene homolog (GLI) signaling pathways, as well as the activation of collagen type I alpha 1 (COL1A1), thereby disrupting glutamine-mediated crosstalk between pancreatic cancer cells (PCCs) and PSCs. This mechanism inhibited cancer cell proliferation and migration while concurrently attenuating PSC activation and function, reducing collagen deposition in the tumor microenvironment, enhancing drug delivery, and potentiating the efficacy of chemotherapeutic agents such as gemcitabine (GEM) (Chakravarthy et al., 2018). In cervical cancer, compared to berberine (another Coptis alkaloid), palmatine exhibits stronger specific interactions with HPV E6 protein, demonstrating higher negative binding energy and lower inhibition constants, which may underlie its anti-metastatic mechanisms (Singh et al., 2021). Furthermore, palmatine prevents prostate cancer cell invasion by suppressing ribosomal protein S6 (rpS6)/NF-κB activation and downstream FLICE-like inhibitory protein (FLIP) gene expression (Hambright et al., 2015).

#### 4.4.3 Angiogenesis inhibition

Palmatine demonstrates significant anti-angiogenic activity. In hepatocellular carcinoma (HCC) studies, Coptis extract containing 4.4% palmatine specifically blocks *de novo* vascular endothelial growth factor (VEGF) protein synthesis by increasing phosphorylation of eukaryotic elongation factor 2 (eEF2) in HCC cells, thereby exerting anti-angiogenic effects (Tan et al., 2014). The canine mammary tumor (CMT)-U27 xenograft mouse model demonstrated that palmatine (50 mg/kg) effectively disrupts tumor vasculature by inhibiting migration and tube formation of canine aortic endothelial cells, showing potential as a canine-specific PI3K inhibitor for treating canine mammary tumors (CMTs) (Yoo et al., 2023).

#### 4.4.4 Immune-inflammatory regulation

Palmatine exhibits marked anti-inflammatory properties. In colon cancer, palmatine (0, 10, 30, 60, 90, and 120 μM) dose-dependently suppresses LPS-induced IL-8 elevation in HT-29 and SW-480 colorectal cancer cells and downregulates intestinal levels of IL-1α, IL-1β, IL-8, G-CSF, and GM-CSF in Apc^Min/+^ mice, ameliorating dysplasia and preventing inflammatory lesions in intestinal tissues (Ma W. K. et al., 2016).

#### 4.4.5 Drug resistance reversal and combination therapy

Palmatine demonstrates potential in overcoming drug resistance and enhancing chemotherapeutic efficacy. Cytotoxicity evaluation studies across four ovarian cancer cell lines (OVCAR-4, OVCAR-8, A2780, and A2780cis) revealed that palmatine maintained activity against cisplatin-resistant A2780 cells (Johnson-Ajinwo et al., 2019; Nakonieczna et al., 2022). When combined with gemcitabine, palmatine significantly enhances growth inhibitory effects in pancreatic stellate cells (PSCs), pancreatic cancer cells (PCCs), and intrinsically gemcitabine-resistant pancreatic cancer cells (Chakravarthy et al., 2018). In prostate cancer, as a key metabolite of Phellodendron amurense bark extract, palmatine synergistically inhibits the rpS6/NF-κB/FLIP signaling axis along with other bioactive metabolites, effectively reducing tumor invasiveness (Hambright et al., 2015). In addition, it can also demonstrate significant antitumor efficacy against various malignancies including gastrointestinal cancers, liver cancer, and soft tissue tumors, through synergistic interactions with active metabolites such as berberine and phellodendrine. Its anticancer mechanisms involve direct induction of tumor cell apoptosis, suppression of cancer cell proliferation, and reduction of tumor cell populations. These synergistic antitumor effects have been validated in multiple studies (He et al., 2022; Nakonieczna et al., 2022; Shinji et al., 2020; Tuzimski et al., 2024), providing an experimental foundation for developing natural alkaloid-based combination antitumor strategies.

#### 4.4.6 Selective toxicity

Palmatine exhibits minimal effects on normal cells. In prostate cancer studies, varying concentrations (0.5, 1, 2.5, 5, and 10 μg/mL) of palmatine demonstrated inhibitory effects on the proliferation of human prostate cancer cell lines (LNCaP, C4-2B, PC-3, and DU145), particularly suppressing the invasive capacity of DU145 and PC-3 cells, while showing no significant impact on non-tumorigenic prostate epithelial cells (Hambright et al., 2015). In breast cancer *in vitro* experiments, palmatine treatment at concentrations ranging from 0.5–100 μg/mL on human estrogen receptor-positive breast cancer cell lines (MCF-7, T47D, and ZR-75–1) and normal human mammary epithelial cells (MCF-10A) revealed IC_50_ values between 5.126 and 5.805 μg/mL, with dose-dependent inhibition of cancer cell proliferation but minimal effects on normal mammary epithelial cells (Grabarska et al., 2021). Similarly, ovarian cancer studies demonstrated low cytotoxicity toward immortalized human ovarian epithelial (HOE) cells (Johnson-Ajinwo et al., 2019).

**FIGURE 5:**
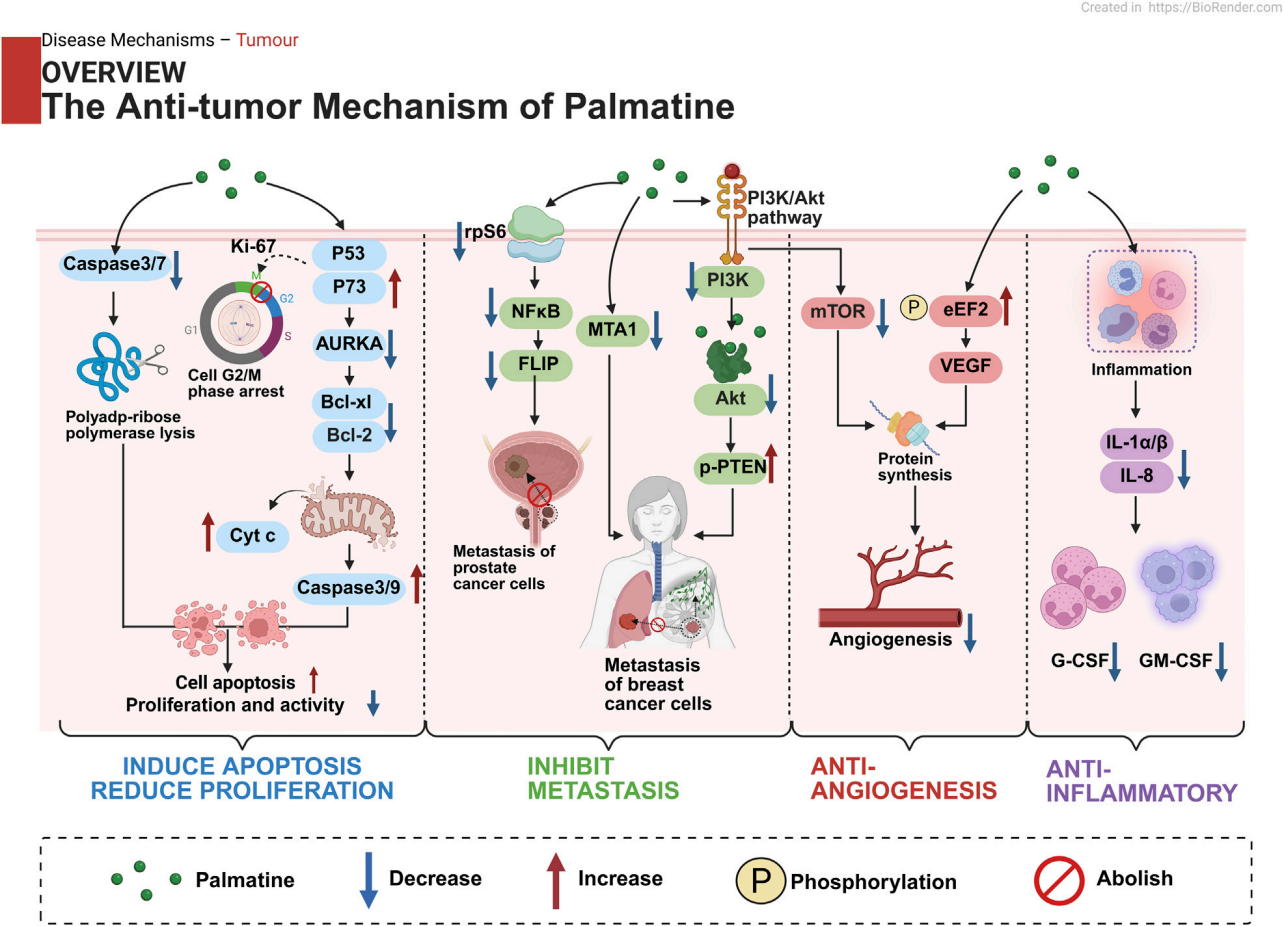
Multi-modal anti-tumor mechanisms of palmatine in cancer therapy: Triggering programmed cell death in malignant cells; Inhibiting cancer cell growth and division; Impeding tumor cell migration and invasion; Disrupting tumor vascularization; Modulating tumor microenvironment.

Collectively, palmatine exerts antitumor effects through multifaceted mechanisms including cell cycle modulation, pro-apoptotic signaling activation, immunoinflammatory suppression, metastasis inhibition, and angiogenesis blockade (Figure 5; Table 4). Its direct cytotoxicity involves AURKA-targeted induction of G2/M phase arrest and caspase cascade-mediated apoptosis. Additionally, palmatine impedes tumor-stromal crosstalk by downregulating metastasis-associated proteins such as MTA1 and COL1A1 while suppressing VEGF-mediated neovascularization. Significant antitumor efficacy has been demonstrated across diverse malignancies, including breast, prostate, colorectal, and ovarian carcinomas. Crucially, palmatine exhibits low selective toxicity toward normal cells (Hambright et al., 2015; Johnson-Ajinwo et al., 2019) and overcomes resistance to conventional chemotherapeutics (Chakravarthy et al., 2018). These attributes—combined with its tumor-selective cytotoxicity and synergistic enhancement of traditional therapies—position palmatine as a promising multimodal anticancer agent, establishing a foundation for natural product-based targeted combination regimens and highlighting its translational advantage for clinical development.

**TABLE 4:** Molecular mechanisms of palmatine in cancer.

Disease model	Study type	Experimental subjects and induction methods	Dose Range/Du ration	Key biological pathways	Molecular and cellular mechanisms of action	References
Colon cancer	*In vitro*	HCT-116, SW480 cells	0, 88, 176, 352, 704 μM; 24 h/48 h/72 h	AURKA kinase	AURKA, Bcl-xl, Bcl2↓ ROS↑; P53, P73, caspase-3, caspase-9↑; cyt.c↑; Ki-67↓; Cancer cell proliferation↓, Cell apoptosis↑	Liu et al. (2020b)
		HT-29 cells	0, 141, 282, 564, 1128 μM; 24 h/48 h/72 h			
	*In vivo*	BALB/c nude mice, HCT-116 cells transplant	33.75, 67.5, 135 mg/kg; 26 days			
Colorectal cancer	*In vivo*	Apc^Min/+^ mice, HFD	10, 20 mg/kg/d; 0–27 weeks	NF-κB, IL-1/IL-8 inflammatory signaling	IL-1α, IL-1β, IL-8↓; G-CSF, GM-CSF↓	Ma et al. (2016b)
	*In vitro*	HT-29 cells, SW-480 cells	0, 10, 30, 60, 90, 120 μM; 48 h			
CMT	*In vivo*	BALB/c and Slc-nu/nu mice, CMT-U27 cells transplant	50 mg/kg; 21 days	PI3K/AKT/mTOR	PI3K, AKT, mTOR↓; p-PTEN↑; Cell death↓	Yoo et al. (2023)
	*In vitro*	CMT-U27 cells	0 μM, 50 μM, 100 μM, 200 μM; 18 h			
BC	*In vitro*	MCF-7, T47D, ZR-75–1, MCF-10A cells	0.5, 1, 2.5, 5, 10, 25, 50, 100 μg/mL; 72 h	Mitochondrial apoptosis	Cancer cell activity and proliferation↓	Grabarska et al. (2021)
	*In vivo*	BALB/c mice, 4T1 cells transplant	1, 5, 10 mg/kg; 28 days	MTA1/p53	MTA1↓; p53↑	Ativui et al. (2022)
PCa	*In vitro*	LNCaP, C4-2B, PC-3, DU145, TRAMPC1 cells	0.5, 1, 2.5, 5, 10 μg/mL; 72 h	rpS6/NF-κB/FLIP signaling	rpS6↓; NF-κB, FLIP↓	Hambright et al. (2015)
OC	*In vitro*	OVCAR-4, OVCAR-8, A2780, A2780cis cells	5.5–7.9 µM; 72 h	Apoptosis	Activation of caspase-3/7↑, Cell apoptosis↑	Johnson-Ajinwo et al. (2019)
OSCC	*In vitro*	CAL27 cells	10, 50, 100, 150, 300 μM; 24 h	PI3K/AKT/mTOR, Apoptosis	PI3K, AKT, mTOR↓; TNF-α, IL-1β↓; BAX↑, Bcl-2, Bcl-xl↓; Cell apoptosis↑	Li et al. (2025)

AURKA, Aurora kinase A; Bcl-xl, B-cell lymphoma-extra Large; Bcl-2, B-cell lymphoma-2; ROS, reactive oxygen species; caspase, Cysteine-dependent Aspartate-specific Protease; cyt.c, Cytochrome c; Ki-67, antigen identified by monoclonal antibody Ki-67; HFD, High-Fat Diet; IL-1α/1β/8, Interleukin 1α/1β/8; G-CSF, Granulocyte Colony-Stimulating Factor; GM-CSF, Granulocyte-Macrophage Colony-Stimulating Factor; CMT, canine mammary gland tumor; PI3K, Phosphoinositide 3-Kinase; AKT, Protein Kinase B; mTOR, mechanistic target of rapamycin; PTEN, Phosphatase and Tensin Homolog Deleted on Chromosome 10; BC, breast cancer; MTA1, Metastasis-Associated Protein 1; PCa, Prostatic Cancer; rpS6, Ribosomal Protein S6; NF-κB, nuclear factor-κB; FLIP, FLICE-like Inhibitory Protein; OC, ovarian cancer; OSCC, oral squamous cell carcinoma; BAX, B-cell lymphoma 2 (Bcl-2)-associated x protein.

### 4.5 Other diseases

#### 4.5.1 Myocardial remodeling

Palmatine demonstrates multi-target therapeutic potential in cardiac hypertrophy and fibrosis by modulating key signaling pathways. In the isoproterenol (ISO)-induced rat model of myocardial hypertrophy, palmatine treatment groups (25 mg/kg and 50 mg/kg) selectively suppressed histone deacetylase histone Deacetylase 2 (HDAC2) expression compared to the model group. Through modulation of the AKT/mTOR pathway, palmatine reduced transcriptional levels of myocardial hypertrophy markers ANP and BNP, while inhibiting the hyperactivation of the HDAC2-KLF4/INPP5F pathway. This led to a dose-dependent restoration of downstream effector genes KLF4 and INPP5F, significantly improving cardiac hypertrophy parameters and alleviating myocardial hypertrophy (Yuan et al., 2017). Regarding myocardial fibrosis, administration of 25 mg/kg or 50 mg/kg palmatine in an ISO-induced mouse model of myocardial fibrosis demonstrated that palmatine inhibited cardiac fibroblast (CF) activation by suppressing the STAT3 signaling pathway, reduced TGF-β and IL-6 protein expression, and improved cardiac structure and function (Lin C. H. et al., 2024). Notably, in zebrafish models of aristolochic acid (AA)-induced heart failure, palmatine activates EGFR signaling and inhibitor of NF-κB kinase β (IKBKB) enzyme activity, restoring cardiac morphology and blood supply while mitigating AA-induced ATPase inhibition. This improves myocardial energy metabolism and reduces inflammatory responses (Hu et al., 2024). These coordinated multi-pathway effects establish a therapeutic network encompassing hypertrophy inhibition, fibrosis blockade, and metabolic improvement, positioning palmatine as a potential pleiotropic agent for myocardial remodeling.

#### 4.5.2 Acute lung injury

Palmatine exerts multi-target protection in acute lung injury (ALI) through synergistic regulation of macrophage polarization, oxidative stress, and pyroptosis pathways. For inflammation control, it significantly reduces LPS-induced M1 macrophage polarization in mice and RAW264.7 cells by inhibiting nicotinamide phosphoribosyltransferase (NAMPT)/Toll-like Receptor 2 (TLR2)/C-C Chemokine Receptor 1(CCR1) signaling, while promoting M2 phenotype expression and downregulating NLRP3 inflammasome activity (Wang et al., 2024). This significantly alleviated inflammatory cell infiltration and decreased TNF-α, CXCL-1, and CXCL-2 levels in bronchoalveolar lavage fluid (Song et al., 2022). Additionally, palmatine blocks TGF-β-Activated Kinase 1 (TAK1)/TAK1-Binding Protein 1 (TAB1) phosphorylation and subsequent p38 Mitogen-Activated Protein Kinase (MAPK)/NF-κB pathway activation (Song et al., 2022). Palmatine (30 mg/kg and 90 mg/kg) antioxidant effects are mediated via Nrf2 pathway activation - in particulate matter (PM)-induced ALI models, palmatine reduces intracellular ROS and inhibits PM-triggered NLRP3-dependent pyroptosis in BEAS-2B cells (Zuo et al., 2024). This multi-layered regulatory network targeting “inflammation-oxidative stress-cell death” pathways, combined with its capacity to reduce neutrophil infiltration and restore lung architecture, provides a solid molecular basis for palmatine as an ALI therapeutic agent.

#### 4.5.3 Malaria

As a representative isoquinoline alkaloid, palmatine exerts potent antimalarial effects through multiple pathways. *In vitro* studies demonstrate significant antiplasmodial activity against Plasmodium falciparum strains Pf3D7 and PfRKL-9 (IC_50_ < 1 μg/mL). Molecular docking reveals palmatine forms stable complexes with plasmodial aminopeptidase N, disrupting protein synthesis (Gogoi et al., 2025). Notably, palmatine enhances therapeutic efficacy in drug combinations. When co-administered with methyl gallate (at a 3:2 ratio) and piperine (as a bioenhancer) in P. berghei NK65-infected mice, it potently inhibits β-hematin formation (IC_50_ = 0.73 μg/mL), causing lethal heme accumulation in parasite food vacuoles (Adegunloye and Adebayo, 2024). Modern research identifies protoberberine alkaloids as rapid-acting antiplasmodials active against all erythrocytic stages of malaria parasites (Erhunse et al., 2024). These findings highlight the potential of alkaloid synergism for developing novel multi-stage antimalarial regimens.

#### 4.5.4 Intervertebral disc degeneration and urticaria

Palmatine exhibits multi-tissue protective effects through specific activation of autophagy pathways in both intervertebral disc degeneration (IDD) and chronic spontaneous urticaria (CSU). In IDD rat models, palmatine upregulates transcription factor EB (TFEB)-mediated autophagy to inhibit endoplasmic reticulum stress (ERS), thereby reducing nucleus pulposus (NP) cell apoptosis and suppressing extracellular matrix (ECM) degradation (Yu et al., 2025). In skin lesions of ovalbumin (OVA)-induced chronic spontaneous urticaria (CSU) rat models, palmatine activates the liver kinase B1 (LKB1)/AMPK autophagy pathway to ameliorate skin damage. It modulates the expression of LC3, Beclin-1, p-LKB1, p-AMPK, Atg5, and Atg12 while inhibiting P62 and p-p70S6K1 expression. These effects alleviate pruritus and mast cell degranulation, reduce neutrophil recruitment, and decrease the release of inflammatory mediators and cytokines, ultimately activating autophagy and improving inflammatory responses to protect against CSU (Xiao et al., 2023). Although acting on different tissues, palmatine achieves its protective effects in both diseases through core autophagy pathways TFEB or AMPK. This shared mechanism of action targeting fundamental autophagy processes provides a molecular basis for the precision medicine applications of palmatine.

Centered on its core actions of inflammation modulation, autophagy regulation, and oxidative stress control, palmatine demonstrates broad-spectrum therapeutic efficacy across cardiovascular, respiratory, infectious, and immune-related disorders (Table 5). In cardiovascular protection, it attenuates myocardial hypertrophy by inhibiting HDAC2-KLF4 signaling, blocks STAT3-mediated cardiac fibroblast activation, and restores cardiac metabolism through EGFR/IKBβ pathway activation. For acute lung injury, palmatine resolves inflammation via NAMPT/TLR2 suppression, TAK1/TAB1-NF-κB inhibition, and Nrf2-mediated antioxidant defense. Furthermore, Furthermore, palmatine mediates a unified therapeutic response through the TFEB/AMPK-autophagy axis, concurrently inhibiting extracellular matrix (ECM) degradation in intervertebral disc degeneration and suppressing mast cell degranulation in urticaria. This mechanistic synergy underscores autophagy modulation as a central therapeutic strategy, solidifying palmatine’s trans-system polypharmacological potential.

**TABLE 5:** Molecular mechanisms of palmatine in other disease systems.

Disease model	Study Type	Experimental subjects and induction methods	Dose Range/Du ration	Key biological pathways	Molecular and cellular mechanisms of action	References
Cardiac hypertrophy	*In vivo*	Wistar rat, ISO	25, 50 mg/kg; 14 days	HDAC2-KLF4/INPP5F signaling	ANP, BNP↓; HDAC2↓, KLF4↑, INPP5F↑	Yuan et al. (2017)
Myocardial fibrosis	*In vivo*	C57BL/6 mice, ISO	25, 50 mg/kg; 7 days	STAT3	STAT3↓, TGF-β1↓, IL-6↓, COL1A1↓, COL3A1↓, α-SMA↓, CTGF↓	Lin et al. (2024b)
	*In vitro*	CFs cells, TGF-β1 (10 ng/mL)	5, 10 μM; 24 h			
ALI	*In vivo*	C57BL/6 mice, PM (4 mg/kg)	50, 100 mg/kg; 48 h	Nrf2/HO-1; NLRP3 inflammasome; ROS/pyroptosis	Nrf2↑, NLRP3↓, ROS↓	Zuo et al. (2024)
	*In vitro*	BEAS-2B cells, PM (200 μg/mL)	80 μM; 24 h			
	*In vitro*	BMDMs, AMs	3, 10, 30 μM; 30 min	TAK1/TAB1; NF-κB; p38 MAPK	BALF↓; NO↓; TNF-α, CXCL-1, CXCL-2↓; iNOS↓; p-p38 MAPK, NF-κB p65 phosphorylation↓, IκBα↑; p-TAK1, p-TAB1↓	Song et al. (2022)
	*In vivo*	C57BL/6 mice, LPS (5 mg/kg)	10, 30, 90 mg/kg; 6 h			
Urticaria	*In vivo*	SD rat, OVA	40 mg/kg; 9 days	Autophagy; Inflammatory cytokine signaling	LC3, Beclin-1, p-LKB1, p-AMPK↑; Atg5, Atg12, Atg5-Atg12↑; P62, p-p70S6K1↓	Xiao et al. (2023)
IDD	*In vivo*	SD rat, acupuncture	50, 100 mg/kg; 4 weeks	TFEB-autophagy; ER stress	BAX, CHOP, Cleaved caspase-3↓; Collagen II, LC3, TFEB↑; ROS↓; Cell apoptosis↓, Bcl-2↑; ADAMTS5, MMP-13↓; CHOP, ATF4, p-eIF2α, GRP78, p-PERK↓; Beclin-1, LC3-II↑; p62↓	Yu et al. (2025)
	*In vitro*	NP cells, TBHP (30 µM)	80 μM; 48 h			
Malaria	*In vivo*	Swiss female albino mice, Plasmodium berghei NK65	6.25, 12.5, 25, 50, 100 mg/kg; 3 days	Heme detoxification	Beta-hemoglobin↓	Adegunloye and Adebayo (2024)

ISO, isoproterenol; ANP, atrial natriuretic peptide; BNP, brain natriuretic peptide; HDAC2, Histone Deacetylase 2; KLF4, Krüppel-like Factor 4; INPP5F, Inositol Polyphosphate-5-Phosphatase F; TGF-β1, Transforming Growth Factor-beta 1; STAT, signal transducer and activator of transcription; IL, interleukin; COL1A1, Collagen Type I Alpha 1 Chain; COL3A1, Collagen Type III, Alpha 1 Chain; α-SMA, α-Smooth Muscle Actin; CTGF, connective tissue growth factor; ALI, acute lung injury; PM, particulate matter; Nrf2, Nuclear factor erythroid 2-related factor 2; NLRP3, Nod-like Receptor Protein 3; ROS, oxygen species; AMs, Alveolar Macrophages; BALF, bronchoalveolar lavage fluid; NO, nitric oxide; CXCL, C-X-C motif chemokine ligand; iNOS, inducible nitric oxide synthase; MAPK, Mitogen-Activated Protein Kinase; NF-κB, nuclear factor-κB; IκBα, Inhibitor of kappa B-α; TAK1, TGF-β-Activated Kinase 1; TAB1, TAK1-Binding Protein 1; SD, Sprague-Dawley; OVA, ovalbumin; LC3, Light Chain 3; LKB1, Liver Kinase B1; AMPK, AMP-activated protein kinase; Atg, Autophagy-related protein; IDD, intervertebral disc degeneration; BAX, B-cell lymphoma 2 (Bcl-2)-associated x protein; CHOP, C/EBP, homologous protein; caspase, Cysteine-dependent Aspartate-specific Protease; TFEB, transcription factor EB; Bcl-2, B-cell lymphoma-2; ADAMTS5, a Disintegrin and Metalloproteinase with Thrombospondin Motifs 5; MMP, matrix metalloproteinase; ATF4, Activating Transcription Factor 4; eIF2α, Eukaryotic Initiation Factor 2 Alpha; GRP78, Glucose Regulated Protein 78 kDa; PERK, Protein Kinase R-like Endoplasmic Reticulum Kinase.

## 5 Pharmacological actions of palmatine: new perspectives

### 5.1 Classical pharmacological effects

Palmatine, a prototypical isoquinoline alkaloid with a longstanding ethnomedicinal history, demonstrates pharmacological effects extending far beyond its traditional applications. Contemporary research has systematically characterized its polypharmacological properties, revealing unique yet interconnected biological activities mediated through pathway-selective mechanisms.

#### 5.1.1 Anti-inflammatory effects

As a principal anti-inflammatory metabolite in traditional Chinese botanical drug extracts (Li et al., 2015), palmatine demonstrates significant anti-inflammatory effects in various disease models particularly inflammatory bowel disease and rheumatoid arthritis. In colitis models, palmatine exerts anti-inflammatory actions by promoting the accumulation and functional enhancement of tolerogenic dendritic cells (DCs) while facilitating regulatory T cell differentiation (Huang et al., 2024). In DSS-induced experimental colitis, palmatine alleviates intestinal inflammation through inhibition of M1 macrophage polarization, reduces levels of pro-inflammatory cytokines TNF-α, IL-1β, IL-6 and IL-8, and enhances tight junction protein ZO-1 expression, thereby restoring intestinal barrier dysfunction (Ji et al., 2024). The anti-inflammatory mechanisms primarily involve suppression of key inflammatory signaling pathways NF-κB, MAPK, Nrf2 and PI3K/AKT and NLRP3 inflammasome activation, leading to decreased pro-inflammatory cytokine production, inhibition of glial cell and macrophage activation, and modulation of immune cell functions (Cheng et al., 2022b; Ji et al., 2024; Ma et al., 2021).

#### 5.1.2 Antioxidant activity

The antioxidant effects of palmatine are intrinsically linked to its anti-inflammatory properties, playing a pivotal role in mitigating oxidative stress-associated damage, which is implicated in various chronic diseases including diabetes, neurodegenerative disorders, and cardiovascular diseases. Its antioxidant mechanisms primarily involve scavenging free radicals and modulating redox homeostasis by disrupting oxidative cascade reactions in cell membranes and interacting with reactive oxygen and nitrogen species (Nwabueze et al., 2022). Furthermore, palmatine enhances cellular antioxidant defenses through activation of the Nrf2/HO-1 signaling pathway, leading to the upregulation of antioxidant enzymes such as SOD, catalase (CAT), and glutathione peroxidase (GSH-Px) (Cheng et al., 2022a; Jia et al., 2021; Okechukwu et al., 2021; Pei et al., 2023; Tang C. et al., 2021; Wang et al., 2023). This process effectively eliminates excessive reactive oxygen species (ROS), reduces β-amyloid (Aβ) toxicity (Lin et al., 2022), and downregulates the expression of Kelch-like ECH-associated protein 1 (Keap-1) (Pei et al., 2023; Wang et al., 2023), thereby suppressing oxidative stress and protecting cells from damage. Additionally, palmatine confers cytoprotection against oxidative injury by inhibiting NLRP3 inflammasome activation, reducing the levels of NLRP3, ASC, IL-1β, and caspase-1 proteins. This mechanism not only enhances cell viability but also promotes cellular recovery and regeneration, underscoring its critical role in neuroprotection (Cheng et al., 2022a).

#### 5.1.3 Anti-fibrotic effects

Palmatine has demonstrated promising therapeutic potential in mitigating fibrosis across various organs, including the liver and heart. It directly targets key fibrotic proteins and modulates TGF-β1/STAT3 signaling pathways, thereby suppressing the expression of fibrotic markers such as COL1A1 and α-SMA (Zhang et al., 2023). This mechanism inhibits fibroblast activation and ultimately attenuates tissue fibrosis (Lin S. et al., 2024). Notably, contemporary studies reveal that palmatine additionally regulates liver-gut axis metabolism by increasing short-chain fatty acids (butyrate/propionate) and correcting amino acid metabolic disturbances. It reverses the depletion of glutamine, taurine, isoleucine, and aspartate while improving intestinal barrier function and microenvironment homeostasis (Qin et al., 2023). These effects collectively establish a multi-target network encompassing “anti-fibrotic action-metabolic regulation-microbial balance.

#### 5.1.4 Antimicrobial and antiparasitic activity

Palmatine exhibits broad-spectrum antimicrobial activity against various pathogens, including bacteria (*S. aureus*, *H. pylori*, and *Escherichia coli*) and parasites (Plasmodium spp.) (Tarabasz and Kukula-Koch, 2020). Its antibacterial mechanisms primarily involve disrupting cell walls and membranes, increasing membrane permeability, releasing functional proteins, damaging DNA structure, and modulating host immune cell responses. As a natural aggregation-induced emission (AIE) photosensitizer, palmatine demonstrates potent photodynamic antibacterial and anti-biofilm activities. Notably, it shows a minimum inhibitory concentration (MIC) of 40 μM against *Listeria* monocytogenes, with 80 μM (minimum bactericidal concentration, MBC) achieving >99.9% bactericidal efficiency after photodynamic treatment (Peng et al., 2024). Traditional medicine has long employed palmatine for gastrointestinal infections like dysentery and diarrhea. Modern pharmacological studies validate this ethnomedical use, demonstrating its dose-dependent inhibition of *H. pylori* urease (HPU) and jack bean urease (JBU) in gastric ulcer and gastritis treatment (Zhou et al., 2017), thereby suppressing *H. pylori* growth and alleviating gastritis.

Furthermore, palmatine exhibits potent antimalarial activity against both chloroquine-sensitive (Pf3D7, IC_50_ = 0.19 ± 0.05 μg/mL) and chloroquine-resistant (PfRKL-9, IC_50_ = 0.38 ± 0.05 μg/mL) Plasmodium falciparum strains (Gogoi et al., 2025), highlighting its therapeutic potential for malaria. These findings underscore palmatine’s multifunctionality as a urease inhibitor, photodynamic antimicrobial agent, and antimalarial metabolite, supported by its low MIC/MBC values and multi-target effects.

However, despite promising *in vitro* results, palmatine’s clinical translation is limited by insufficient *in vivo* mechanistic studies and pharmacokinetic characterization. Its safety profile under prolonged or high-dose antimicrobial conditions remains understudied, and evaluations in polymicrobial or drug-resistant infection environments are lacking. Given its potential synergistic advantages, future research should investigate combinations with existing antibiotics—particularly against multidrug-resistant (MDR) pathogens—and elucidate host-pathogen interactions and immunomodulatory mechanisms in infection models.

#### 5.1.5 Additional pharmacological effects

Furthermore, palmatine demonstrates antitumor, hypoglycemic, hypolipidemic, and hepatoprotective activities. Recent studies reveal that palmatine exhibits multiple anticancer effects in various tumor models, including anti-proliferative, pro-apoptotic, and anti-angiogenic properties, primarily mediated through AURKA kinase, p53, PI3K/AKT, and VEGF pathways (see Section 4.4; Table 4). Regarding its hypoglycemic and hypolipidemic effects, palmatine modulates glucose and lipid metabolism via endoplasmic reticulum stress, insulin signaling, oxidative stress, and purine metabolism, involving mechanisms such as the IRS1/AKT2/FOXO1 axis, PPARα-CYP7A1 pathway, and ASBT-mediated bile acid metabolism, thereby serving as a key regulator in restoring insulin sensitivity and lipid homeostasis (Chen et al., 2021; Ning et al., 2020; Nwabueze et al., 2022; Xia et al., 2022). The broad pharmacological properties of palmatine in traditional medicine have been mechanistically validated by modern pharmacological research, providing a solid foundation for its development as a multi-target therapeutic agent.

In summary, as a multi-target natural isoquinoline alkaloid, palmatine has been extensively studied for its diverse pharmacological properties (Tarabasz and Kukula-Koch, 2020). Its anti-inflammatory, antioxidant, anti-fibrotic, antimicrobial, anticancer, hypoglycemic, and hypolipidemic activities underscore its therapeutic versatility and potential clinical applications. Among these, anti-inflammatory and antioxidant effects represent the core mechanisms underlying its therapeutic efficacy across various pathological conditions. Substantial evidence indicates that palmatine modulates key signaling pathways such as NF-κB, Nrf2/HO-1, PI3K/AKT, MAPK, AMPK/mTOR, TGF-β1/Smad, and STAT3, thereby regulating inflammation, oxidative stress, apoptosis, autophagy, and fibrotic remodeling. These findings highlight its promise as a multifunctional therapeutic candidate for chronic inflammatory diseases, metabolic disorders, fibrosis, infections, and malignancies. However, given its broad activity spectrum, disease-specific investigations are warranted to identify critical mechanistic nodes driving its therapeutic effects and elucidate potential adverse cross-reactivities. Particularly in the era of precision medicine and integrative approaches, sustained mechanistic research, pharmaceutical development, and translational studies will be pivotal in establishing palmatine as a clinically viable therapeutic agent.

### 5.2 Development of novel drug formulations

Recent advances in nano-delivery technology have expanded innovative applications of palmatine as a drug design scaffold, particularly demonstrating remarkable advantages in tumor immunotherapy and central nervous system disorders. In structural modification studies, researchers chemically functionalized the O-9 position of palmatine to conjugate with anti-PD-L1 peptides containing disulfide linkers, constructing a glutathione (GSH)-responsive prodrug system. This system enables tumor microenvironment-selective drug release, simultaneously blocking extracellular PD-L1 while activating intracellular PD-L1 degradation mechanisms, thereby significantly enhancing antitumor immune responses (Zeng Z. et al., 2024).

Extending to neurological diseases, extracellular vesicles (EVs) have emerged as promising delivery platforms due to their low immunogenicity, biodegradability, and innate blood-brain barrier (BBB) penetration capability. Studies demonstrate that a microglia-derived exosome-based co-delivery system (Exos-Ber/Pal) encapsulating both berberine and palmatine outperforms free drugs. Leveraging the exosomal lipid bilayer structure and transmembrane proteins, this system achieves approximately 3.2-fold higher drug accumulation in the hippocampus and cortex, with 48-h cumulative release rates of 72.59% ± 7.46% and 72.88% ± 9.90%, respectively, while enhancing brain-targeted delivery and therapeutic efficacy (Zhao et al., 2023). Further development yielded a transferrin-modified extracellular vesicle system (Tf-hEVs-Ber/Pal), which utilizes transferrin receptor (TfR)-mediated transcytosis to cross the BBB and precisely target microglia. In an Alzheimer’s disease mouse model, this delivery system effectively cleared β-amyloid (Aβ) aggregates, modulated neuroinflammatory microenvironments, and significantly improved cognitive function and learning capacity (Zhou et al., 2025).

While current nano-delivery systems offer promising avenues for the clinical application of palmatine, its poor aqueous solubility and metabolic stability significantly limit its broader implementation in nanocarrier systems. Moreover, the non-specific biodistribution of exosomes to off-target organs such as the heart and kidneys may lead to systemic toxicity or adverse effects, necessitating further in-depth investigation. Future advancements in palmatine formulation development should concentrate on integrating nanotechnology with biomimetic approaches that exploit the inherent biological properties of extracellular vesicles to improve targeted drug delivery. Additionally, exploring combination therapies through co-delivery systems that pair palmatine with other bioactive molecules could enhance multi-pathway therapeutic effects while increasing drug loading efficiency, potentially enabling the transformation of palmatine from a conventional single-agent therapy to a modern precision-targeted pharmaceutical.

### 5.3 Innovative applications of metabolites

Palmatine’s oxidized metabolites exhibit superior therapeutic potential compared to the parent metabolite. In ulcerative colitis (UC), 8-oxypalmatine (OPAL) demonstrates dual mechanisms: inhibiting M1 macrophage infiltration while promoting M2 polarization, modulating inflammatory cytokines and anti-inflammatory factors, while upregulating tight junction proteins to restore intestinal barrier integrity (Huang et al., 2025). OPAL also activates the Nrf2 pathway and suppresses NLRP3 inflammasome activation more effectively than palmatine (Cheng et al., 2022b).

In hyperuricemic nephropathy, 9-hydroxy-8-oxypalmatine (9-OPAL) shows enhanced renoprotection by suppressing URAT1/GLUT9 expression, restoring OAT1 activity, reducing XOD and ADA enzymatic activity and serum markers like CRE, BUN and UA, while inhibiting NLRP3 inflammasome components (Wu et al., 2024). Molecular docking confirms 9-OPAL’s high affinity for XOD and urate transporters.

Notably, structural modifications of palmatine have significantly expanded its therapeutic applications. The 8-oxo-13-substituted dihydropalmatines enhance antibacterial activity by activating X-box binding protein 1 (XBP1) transcriptional activity (Song et al., 2018). The novel oxo-palmatine derivative 2q targets hyperpolarization-activated cyclic nucleotide-gated channel 2 (HCN2) to reduce Aβ deposition and Tau protein phosphorylation, thereby improving Alzheimer’s disease pathological features (Pang et al., 2025). Additionally, palmatine derivative 2v exerts antithrombotic effects through dual mechanisms: binding protein kinase G to improve vasodilator-stimulated phosphoprotein phosphorylation while simultaneously inhibiting AKT phosphorylation via phosphatidylinositol 3-kinase interaction, ultimately reducing platelet aggregation (Fan J. H. et al., 2022). These findings not only reveal the unique advantages of palmatine metabolites but also provide new directions for structure-based optimization of natural products.

Structurally optimized oxidative metabolites of palmatine have significantly expanded its therapeutic boundaries, demonstrating superior efficacy compared to the parent metabolite in treating ulcerative colitis, hyperuricemic nephropathy, neurodegenerative diseases, and thrombosis prevention. However, the structure-activity relationships between these metabolites and their molecular targets such as XOD and HCN2 remain to be systematically elucidated. Current investigations are predominantly limited to animal models, highlighting the need to advance toward humanized disease model validation and early-stage clinical trials. Further comprehensive studies are imperative to bridge this translational gap and facilitate clinical application.

### 5.4 Synergistic therapeutic strategies

Contemporary studies increasingly focus on palmatine as a key active metabolite in traditional Chinese medicine formulations, elucidating its synergistic mechanisms with other metabolites across disease progression. These investigations reveal palmatine’s remarkable advantages in enzyme inhibition, target modulation, and delivery optimization. Pharmacokinetic analysis of seven metabolites in the Dahuang Fuzi Decoction using UPLC-ESI-MS/MS demonstrated palmatine’s superior absorption profile in ulcerative colitis rat models, exhibiting higher bioavailability (C_max_ = 269.70 ± 22.37 ng/mL, T_max_ = 0.25 ± 0 h), prolonged elimination half-life (t_1/2_ = 5.65 ± 2.22 h), and greater systemic exposure (AUC_(0–24)_ = 1851.90 ± 93.77 ngh/mL, and AUC_(0– ∞ )_ = 1979.52 ± 131.22 ngh/mL) compared to other alkaloids (Yang et al., 2017).

The quaternary ammonium structure of palmatine confers unique pharmacodynamic synergies. When combined with berberine, palmatine exhibits mixed-competitive inhibition of acetylcholinesterase (combination index <1), reducing berberine’s IC_50_ from 0.52 μM to 0.17 μM and significantly enhancing cholinergic neurotransmission (Mak et al., 2014) (Table 6). Conversely, palmatine’s competitive binding to hOCT1 may reduce intestinal absorption of co-administered cationic drugs (Zhang et al., 2022). In benign prostatic hyperplasia (BPH), the Ber/Pal combination moderately inhibits key inflammatory targets 5-lipoxygenase (LOX-5) and cyclooxygenase-2 (COX-2), ameliorating BPH pathology (Wang et al., 2021). Notably, in APP/PS1 mouse models, the combined use of Ber/Pal within an exosomal delivery system (Exos) achieved enhanced brain targeting (drug loading efficiency: Ber 12.2%/Pal 15.2%), concurrently promoting neuronal repair, modulating neuroinflammation, inhibiting Aβ plaque formation, and suppressing microglial activation (Zhao et al., 2023). The composite alkaloid formulation (berberine [BBR], coptisine [COP], palmatine, epiberberine [EPI], and jatrorrhizine [JAT]) demonstrates remarkable synergistic effects. Palmatine-containing combinations more effectively reduce lipid and cholesterol accumulation in HepG2 cells through coordinated upregulation of LDL receptor and CYP7A1 expression with concurrent HMGCR suppression (Kou et al., 2016). When combined with baicalein and triptolide, palmatine synergistically modulates macrophage metabolism via the HIF-1α pathway, promoting M2 polarization for ulcerative colitis treatment (Zhang et al., 2025). In antimalarial therapy, a 3:2 ratio of palmatine to methyl gallate (with piperine adjuvant) shows superior β-hematin formation inhibition (Table 6), inducing toxic accumulation in parasite digestive vacuoles (Adegunloye and Adebayo, 2024).

**TABLE 6:** Synergistic effects and pharmacological interactions of palmatine with other drugs or natural metabolites: Comparative IC_50_ values and mechanistic Overview.

Pharmacological activity	Drug/Combination	IC_50_ value	Key targets	Synergistic effect	Combination effect	References
Acetylcholinesterase Inhibition	Palmatine	0.46 ± 0.013 μM	AChE	↑AChE affinity 3-fold	Synergistic (∼3-fold enhancement, CI < 1)	Mak et al. (2014)
	Berberine	0.52 ± 0.042 μM				
	Plamatine + Berberine	0.17 ± 0.023 μM				
Plasmodium β-Hematin Formation Inhibition	Palmatine	0.82 μg/mL	β-Hematin formation	↓IC_50_ to 0.73 μg/mL (vs. 0.82 μg/mL alone) ↑Heme accumulation in parasites ↓Parasite rate (>40%)	Synergistic (3:2 ratio)	Adegunloye and Adebayo (2024)
	Methyl Gallate	2.56 μg/mL				
	Chloroquine	0.74 μg/mL				
	Plamatine + Methyl Gallate	0.73 μg/mL				
Anti-Proliferation in Breast Cancer Cells (T47D, MCF-7, ZR-75–1)	Palmatine	(MCF7) 5.194 ± 1.492 μg/mL (T47D) 5.805 ± 2.048 μg/mL (ZR-75–1) 5.126 ± 1.774 μg/mL	Apoptosis, cell proliferation	↓Proliferation: IC_50 exp_: 1.001 ± 0.279 μg/mL, IC_50 add_: 2.603 ± 0.901 μg/mL (ZR-75–1) ↑Early apoptotic cells: MCF7: ≥70%, T47D, ZR-75–1: ≈50% ↑Cell Death Rate: MCF7: 53.21%, T47D: 60.48%, ZR-75–1: 68.06% (with 1/2 IC_50_ PAL +1/2 IC_50_ DOX)	Synergistic (MCF7) and additive (T47D, ZR-75–1)	Grabarska et al. (2021)
	Doxorubicin	(MCF7) 0.104 ± 0.029 μg/mL (T47D) 0.089 ± 0.020 μg/mL (ZR-75–1) 0.080 ± 0.028 μg/mL				
	Palmatine + Doxorubicin	(MCF7) 2.507 ± 0.435 μg/mL (T47D) 1.942 ± 0.411 μg/mL (ZR-75–1) 1.001 ± 0.279 μg/mL				
Inhibition of pancreatic stellate cell proliferation	Palmatine	75 μg/mL	GLI, glutamine metabolism	↓IC_50_: 0.1 µM GEM + 25 μg/mL PAL = 75 μg/mL PAL or 0.25 µM GEM (alone) ↓Proliferation of pancreatic cancer cells ↑Apoptosis of pancreatic cancer cells	Synergistic (CI < 0.5)	Chakravarthy et al. (2018)
	Gemcitabine	25 µM				
	Palmatine + Gemcitabine	0.1 µM GEM + 25 μg/mL PAL				

IC_50_, half maximal inhibitory concentration; AChE, acetylcholinesterase; CI, composite index; IC_50exp_, Experimental IC_50_ value; IC_50 add,_ Theoretical additive IC_50_ value; PAL, palmatine; DOX, doxorubicin; GLI, Glioma-associated oncogene homolog; GEM, gemcitabine.

Beyond its synergistic effects with natural bioactive metabolites, palmatine has demonstrated significant therapeutic potential when combined with semi-synthetic pharmaceuticals, achieving notable breakthroughs in combination therapies. In anticancer regimens, palmatine overcomes multidrug resistance by suppressing survivin, restoring gemcitabine (GEM) sensitivity in pancreatic cancer cells. Notably, combination therapy with reduced doses (25 μg/mL palmatine + 0.1 μM GEM) achieves equivalent proliferation inhibition to monotherapy with 75 μg/mL palmatine or 0.25 μM GEM (Chakravarthy et al., 2018) (Table 6). In MCF7, T47D and ZR-75–1 breast cancer cells, co-administration of half IC_50_ doses of palmatine and doxorubicin induces 53.21%, 60.48%, and 68.06% cell mortality respectively, demonstrating enhanced drug-mediated cytotoxicity (Grabarska et al., 2021) (Table 6). These findings not only provide compelling evidence supporting the scientific basis of natural metabolite synergism but also establish a critical foundation for developing low-dose, high-efficacy combination therapies. Moving forward, research should prioritize clinical investigations focusing on synergistic mechanisms, with particular emphasis on palmatine-berberine combinations for treating metabolic-neurological comorbidities such as diabetes-associated depression, and palmatine-chemotherapy conjugates designed to overcome tumor drug resistance.

## 6 Safety and toxicity of palmatine

Modern research has confirmed that palmatine exhibits significant dose/concentration-dependent therapeutic effects while maintaining excellent safety profiles. Within therapeutic concentration ranges, palmatine demonstrates minimal cytotoxicity (Ativui et al., 2022; Kwon et al., 2016; Ma W. K. et al., 2016; Xu et al., 2021). Toxicity evaluations in HepG2, RAW264.7, HeLa, and L-02 cells showed cell viability exceeding 70% even at 100 mg/mL concentrations (Xu et al., 2021) (Table 7). In IDD models, 80 μM palmatine significantly improved NP cell viability without observable toxicity (Yu et al., 2025; Zhao et al., 2025). Cardiac myocyte experiments revealed that while high concentrations (10 mM) completely inhibited beating rate and amplitude (IC_50_ = 5.2 mM), cell morphology and quantity remained normal (Zhang M. Y. et al., 2018). Hemolysis and cytotoxicity assays further confirmed palmatine’s safety for blood components and excellent biocompatibility at elevated concentrations (Peng et al., 2024). Sedative-hypnotic studies demonstrated that effective doses of palmatine did not affect PC12 cell viability, enhancing cerebral 5-HT content without interfering with the GABA system (Du et al., 2019). Recent acute toxicity tests established an LD_50_ of 1533.68 mg/kg for palmatine and >5000 mg/kg for its derivative 9-hydroxy-8-oxypalmatine (9-OPAL), with neither showing organ-specific toxicity (Alamzeb et al., 2024; Wu et al., 2024; Yi et al., 2013) (Table 7).

**TABLE 7:** Safety profile of palmatine across biological systems.

Parameter	Test system	Key findings	References
Acute Toxicity	Mice (oral)	LD_50_ = 1533.68 mg/kg; No organ damage at ≤500 mg/kg	Wu et al. (2024)
Cytotoxicity	Normal cells (HepG2, RAW264.7, HeLa, L-02 cells)	>90% viability in L-02; >70% in HepG2/RAW264.7/HeLa at 100 μg/mL; Excellent biocompatibility	Xu et al. (2021)
Cardiotoxicity	Cardiac myocytes	IC_50_ = 5.2 mM (heart rate, amplitude) 10 mM: Complete heartbeat arrest; Cell detachment	Zhang et al. (2018a)
Biocompatibility	L-02, MARC-145 cells	Hemolysis rates: <1% (Concentration = 10, 20, 40, 80, 160, 320 μM)	Peng et al. (2024)
Plasma protein binding rat	SD rat	(Rat plasma): 1.0 μg/mL:71.13% ± 0.49%, 4.0 μg/mL:51.17% ± 0.39%, 10.0 μg/mL:41.81% ± 0.74% (Human plasma): 1.0 μg/mL:74.43% ± 0.09%, 4.0 μg/mL:74.34% ± 0.09%, 10.0 μg/mL:55.50% ± 0.54% Human > Rat; Decreases with conc	Song et al. (2021)

LD_50_, lethal Dose, 50%; IC_50_, half maximal inhibitory concentration.

Notably, palmatine exhibits selective cytotoxicity toward cancer cells while sparing normal cells, maintaining activity even against drug-resistant strains (Grabarska et al., 2021; Johnson-Ajinwo et al., 2019; Nakonieczna et al., 2022). In breast cancer, palmatine treatment dose-dependently inhibited cancer cell viability and proliferation (IC_50_ = 5.126–5.805 μg/mL for MCF-7 cells) without affecting normal mammary epithelial cells (Grabarska et al., 2021). Palmatine demonstrates significant cytotoxic effects in OVCAR-4, OVCAR-8, A2780, and A2780cis ovarian cancer cell lines, while exhibiting minimal cytotoxicity in human ovarian epithelial cells (HOE), with IC_50_ values ranging from 5.5 to 7.9 μM. Notably, no cross-resistance was observed in cisplatin-resistant A2780cis cells (Johnson-Ajinwo et al., 2019), while gastric cancer experiments revealed particularly strong inhibition of ACC-201 cells (IC_50_ = 4.909 ± 0.017 μg/mL), with normal intestinal epithelial cells (YAMC) maintaining viability even at high concentrations (100–120 μM) (Nakonieczna et al., 2022). This selectivity likely stems from palmatine’s unique dual anti-inflammatory and anti-proliferative mechanisms rather than nonspecific cytotoxicity (Ma W. K. et al., 2016). Recent findings also highlight palmatine’s retained activity against drug-resistant cancer cells and potential for bioimaging applications (Johnson-Ajinwo et al., 2019; Xu et al., 2021), suggesting promise for developing theranostic agents. These properties are crucial for evaluating palmatine’s safety and efficacy as a potential therapeutic agent.

In summary, extensive preclinical evaluations in both animal and *in vitro* studies have demonstrated that palmatine exhibits an excellent safety profile at clinically relevant concentrations (1–100 μM), showing well-characterized dose-dependent therapeutic effects with minimal toxicity. Specifically, palmatine maintains >70% viability in both hepatic and epithelial cell lines at concentrations below 100 μg/mL without causing organ-specific histopathological damage, while displaying selective cytotoxicity toward malignant cells over normal cells. The main safety considerations involve transient cardiac suppression observed only at extremely high doses (>5.2 mM, far exceeding therapeutic ranges) and potential drug-drug interactions mediated by organic cation transporters (particularly hOCT1/2) as detailed in Table 7. Special caution should be exercised in patients with hepatic or renal impairment due to palmatine’s primary elimination pathways through hepatic metabolism and renal excretion.

Nevertheless, several challenges remain for its comprehensive clinical application, including insufficient data on long-term toxicity and reproductive safety, as well as poorly defined individualized safety thresholds. Future research directions should focus on concurrent optimization of bioavailability and thorough investigation of safety mechanisms, incorporating advanced approaches such as artificial intelligence-based drug interaction network analysis for clinical contraindication prediction and the development of novel prodrug delivery systems. These advancements will be critical for transforming palmatine from a “relatively safe” agent to a “precisely controllable” therapeutic, thereby establishing a new safety paradigm for the clinical translation of natural products.

## 7 Conclusion and perspectives

### 7.1 Multidimensional therapeutic value of palmatine

Palmatine, an isoquinoline alkaloid with a long history of medicinal use, is primarily derived from traditional Chinese botanical drugs such as *Coptis chinensis Franch.* and *Phellodendron amurense Rupr.* Studies indicate that palmatine represents 4.4% of the total composition in Coptis water extracts, second only to berberine in abundance (Tan et al., 2014), making it one of the most significant active metabolites in natural plant extracts. Classical medical texts extensively documented the therapeutic use of palmatine-containing botanical drugs across multiple disease domains. The *Treatise on Cold Pathogenic Diseases* prescribed Huanglian Jiedu Tang for jaundice. The *Qianjin Fang* recorded the application of *Phellodendron amurense Rupr*. in treating febrile dysentery, while the *Bencao Beiyao* documented the efficacy of *Coptis chinensis Franch*. in managing neurological conditions through its sedative properties. Modern research has not only confirmed these traditional applications through its demonstrated antibacterial and anti-inflammatory effects but has further revealed, via network pharmacology and molecular docking studies, that palmatine exerts multi-target pharmacological mechanisms.

Furthermore, palmatine exhibits a broad-spectrum and intricate molecular mechanism in disease treatment, primarily attributed to its simultaneous targeting of three closely intertwined key processes: anti-inflammatory effects, autophagy activation, and metabolic homeostasis regulation. It demonstrates remarkable efficacy in suppressing inflammatory cascades, particularly by inhibiting NLRP3 inflammasome assembly, blocking NF-κB nuclear translocation, and modulating signaling pathways such as STAT1/CXCL10 and TAK1/TAB1-NF-κB. These mechanisms underpin its shared anti-inflammatory basis across various conditions (Song et al., 2022; Zhou et al., 2024), including intestinal inflammation (restoring ZO-1 tight junction protein expression), neuroinflammation, lung injury, gout, and tumor microenvironment regulation. In autophagy regulation, palmatine significantly enhances mitophagy (reducing LC3-II/p62 degradation) (Lin et al., 2022; Yu et al., 2025) through activation of the AMPK/mTOR pathway and the TFEB/PINK1 signaling axis. This mechanism not only maintains neuronal mitochondrial quality and clears damaged hepatic organelles to improve lipid metabolism (e.g., activating the PPARα-CYP7A1 axis to promote cholesterol conversion) but also contributes to cardiac metabolic recovery (inhibiting STAT3-mediated fibrosis) and protection of the extracellular matrix in intervertebral discs (Lin S. et al., 2024; Ning et al., 2020; Yu et al., 2025). Regarding metabolic balance, palmatine improves insulin sensitivity by coordinating IRS1/AKT2/FOXO1 signaling to enhance GLUT4 membrane translocation (Chen et al., 2021; Lin et al., 2022; Nwabueze et al., 2022; Xia et al., 2022), while also modulating purine metabolism and alleviating oxidative stress and endoplasmic reticulum stress through the antioxidant activity of the Nrf2/HO-1 pathway, thereby comprehensively addressing disorders of glucose and lipid metabolism as well as hyperuricemia. In the field of antitumor therapy, palmatine selectively targets AURKA to induce G2/M phase arrest and caspase-dependent apoptosis, while suppressing tumor metastasis by downregulating MTA1/COL1A1 and inhibiting VEGF-mediated angiogenesis. Additionally, it demonstrates low inherent cytotoxic potential and an ability to overcome chemoresistance (Chakravarthy et al., 2018; Tan et al., 2014).

In conclusion, the research highlights of palmatine include: (1) structural modification and delivery technologies, specifically enhancement of *in vivo* stability through structural modifications (e.g., introduction of lipophilic groups at the O-9 position) and novel delivery systems (e.g., peptide-drug conjugate nanocarriers), leading to improved solubility, membrane permeability, reduced circulatory degradation, and enhanced bioavailability and targeting efficiency; (2) unique bioactivity of metabolites, referring to metabolites such as 8-oxo-palmatine and 9-hydroxy-8-oxo-palmatine exhibit superior efficacy compared to the parent metabolite in certain disease models, offering new directions for natural product-based drug development; (3) synergistic therapeutic potential—demonstrated synergy with other agents (e.g., berberine or chemotherapeutics) enhances therapeutic effects while reducing dosage requirements and mitigating drug resistance. These findings not only deepen the understanding of the multi-target mechanisms of natural products but also provide a theoretical foundation for palmatine-based drug development.

### 7.2 Recommendations for future research

Despite significant progress, several challenges remain in palmatine research. First, its oral bioavailability is low due to the quaternary ammonium structure, which limits intestinal absorption and undergoes substantial first-pass metabolism in the liver. Complex metabolic pathways, such as O-demethylation and hydroxylation, further reduce systemic exposure, constraining the efficacy of oral formulations. Second, mechanistic studies lack depth, most current investigations rely on animal models, with insufficient systematic validation of key targets using multi-omics technologies like spatial metabolomics or gene-editing tools such as CRISPR/Cas9. Third, a translational gap exists, as human pharmacokinetic data remain scarce, and standardized large-scale clinical trials are needed to confirm safety, efficacy, and optimal dosing regimens in specific populations, including metabolic syndrome patients. Additionally, palmatine exhibits notable tissue- and disease-specific effects, yet the dynamic regulatory mechanisms underlying its cross-organ modulation—particularly in traditional Chinese medicine (TCM) formulations—through gut microbiota regulation, for example, reshaping microbial communities and improving intestinal barrier function, and its influence on the brain-gut axis or gut microbiota-intestinal barrier-liver function network remain poorly elucidated and warrant further investigation.

To address these limitations, future investigations should focus on several critical areas to advance palmatine research. First, primary emphasis should be placed on developing highly active metabolites including potency-enhanced 8-oxypalmatine and 9-hydroxy-8-oxypalmatine, innovating structural optimization derivatives through C13 alkylation modifications, and employing biomimetic technologies to design targeted nano-delivery systems via computer-aided drug design approaches such as exosome carriers and pH-responsive liposomes, thereby overcoming oral bioavailability limitations while enhancing targeted accumulation in tumor and inflammatory sites.

Second, comprehensive mechanistic studies should integrate proteomics, metabolomics and artificial intelligence-driven molecular docking to delineate the multi-target, multi-pathway interaction networks of palmatine and its metabolites, with particular focus on elucidating its molecular regulation of gut microbiota-host metabolic crosstalk involving short-chain fatty acid production and bile acid cycling, along with precise modulation of downstream signaling pathways.

Third, clinical translation should be accelerated through phase I/II trials targeting priority indications including ulcerative colitis and Alzheimer’s disease, incorporating therapeutic drug monitoring to establish personalized dosing regimens based on metabolic enzyme phenotypes characterized by CYP2D6 and CYP1A2 polymorphisms.

Fourth, sustainable development should be pursued through green extraction technologies employing natural deep eutectic solvents to achieve synergistic resource efficiency and environmental protection. Collaborative exploration with other natural products should investigate palmatine’s synergistic effects in traditional botanical drug formulations, utilizing network pharmacology to decipher its holistic multi-component, multi-target, multi-pathway mechanisms, thereby facilitating modernization of traditional medicine.

Palmatine research not only pioneers novel clinical applications for isoquinoline alkaloids but also establishes a paradigm for multi-targeted natural product development. By overcoming bioavailability constraints, elucidating mechanisms, and advancing clinical validation, palmatine emerges as a transformative therapeutic strategy for complex diseases, ultimately bridging traditional medicine and modern therapeutics.
